# Acari of Canada

**DOI:** 10.3897/zookeys.819.28307

**Published:** 2019-01-24

**Authors:** Frédéric Baulieu, Wayne Knee, Victoria Nowell, Marla Schwarzfeld, Zoë Lindo, Valerie M. Behan-Pelletier, Lisa Lumley, Monica R. Young, Ian Smith, Heather C. Proctor, Sergei V. Mironov, Terry D. Galloway, David E. Walter, Evert E. Lindquist

**Affiliations:** 1 Canadian National Collection of Insects, Arachnids and Nematodes, Agriculture and Agri-Food Canada, Ottawa, Ontario, K1A 0C6, Canada Agriculture and Agri-Food Canada Ottawa Canada; 2 Department of Biology, Western University, 1151 Richmond Street, London, Ontario, N6A 5B7, Canada Western University London Canada; 3 Royal Alberta Museum, Edmonton, Alberta, T5J 0G2, Canada Royal Alberta Museum Edmonton Canada; 4 Centre for Biodiversity Genomics, University of Guelph, Guelph, Ontario, N1G 2W1, Canada University of Guelph Guelph Canada; 5 Department of Biological Sciences,University of Alberta, Edmonton,Alberta, T6G 2E9, Canada University of Alberta Edmonton Canada; 6 Department of Parasitology, Zoological Institute of the Russian Academy of Sciences, Universitetskaya embankment 1, Saint Petersburg 199034, Russia Zoological Institute of the Russian Academy of Sciences St. Petersburg Russia; 7 Department of Entomology, University of Manitoba, Winnipeg, Manitoba, R3T 2N2, Canada University of Manitoba Winnipeg Canada; 8 University of Sunshine Coast, Sippy Downs, 4556, Queensland, Australia University of Sunshine Coast Queensland Australia; 9 Queensland Museum, South Brisbane, 4101, Queensland, Australia Queensland Museum South Brisbane Australia

**Keywords:** Astigmata, biodiversity assessment, Biota of Canada, DNA barcodes, Endeostigmata, Hydrachnidia, Mesostigmata, mites, Oribatida, Prostigmata, ticks

## Abstract

Summaries of taxonomic knowledge are provided for all acarine groups in Canada, accompanied by references to relevant publications, changes in classification at the family level since 1979, and notes on biology relevant to estimating their diversity. Nearly 3000 described species from 269 families are recorded in the country, representing a 56% increase from the 1917 species reported by Lindquist et al. (1979). An additional 42 families are known from Canada only from material identified to family- or genus-level. Of the total 311 families known in Canada, 69 are newly recorded since 1979, excluding apparent new records due solely to classification changes. This substantial progress is most evident in Oribatida and Hydrachnidia, for which many regional checklists and family-level revisions have been published. Except for recent taxonomic leaps in a few other groups, particularly of symbiotic mites (Astigmata: feather mites; Mesostigmata: Rhinonyssidae), knowledge remains limited for most other taxa, for which most species records are unpublished and may require verification. Taxonomic revisions are greatly needed for a large majority of families in Canada. Based in part on species recorded in adjacent areas of the USA and on hosts known to be present here, we conservatively estimate that nearly 10,000 species of mites occur in Canada, but the actual number could be 15,000 or more. This means that at least 70% of Canada’s mite fauna is yet unrecorded. Much work also remains to match existing molecular data with species names, as less than 10% of the ~7500 Barcode Index Numbers for Canadian mites in the Barcode of Life Database are associated with named species. Understudied hosts and terrestrial and aquatic habitats require investigation across Canada to uncover new species and to clarify geographic and ecological distributions of known species.

## Introduction

With more than 54,000 described and 0.4–1.0 million estimated species worldwide (Zhang 2011, Walter and Proctor 2013), mites are among the most diverse groups of animals on the planet, and one of the least well known. Approximately 10,000 species were estimated to occur in Canada by Lindquist et al. (1979), 80% of which were thought to be unrecorded at the time. Correlated with their taxonomic diversity, mites are ecologically ubiquitous, with habitats ranging from bear fur and bird feathers to leaf domatia, to insect spiracles, and mountain tops to ocean trenches (Lindquist et al. 1979, Lumley et al. 2013, Walter and Proctor 2013). While their minute size has been a key ingredient in their evolutionary success, it has also been an impediment to taxonomic progress. In addition, small size means that mites are often overlooked by researchers other than acarologists, obscuring awareness of their impact on agriculture, forestry, wildlife, human health, and ecosystem services. Mites recycle nutrients in soil and other substrates, and regulate animal, fungal and plant populations as consumers or pathogen vectors (Moore et al. 1988, Hoy 2011). While several are pests that threaten agriculture by feeding on crops and livestock, others protect it as biocontrol agents of pests and weeds (Rosenthal 1996, Gerson et al. 2003). Some mites are also bioindicators of soil and freshwater health (BehanPelletier 1999, Beaulieu and Weeks 2007, Proctor 2007).

Herein, we treat Acari as a single group, following Lindquist et al. (1979) and other major references (e.g., Krantz and Walter 2009). However, the ‘Acari’ may represent two independent lineages of arachnids instead of a single monophyletic clade, a hypothesis supported by recent molecular analyses (e.g., Pepato and Klimov 2015), and possibly by morphology (Lindquist 1984, Dunlop and Alberti 2007). Based on fossil records, the origin of acariform mites dates back at least to the early Devonian (411 mya), possibly as detritivore-scavengers living in the interstices of beach sand or soil, after land was colonized by ancestral arachnids (Schaefer et al. 2010, Walter and Proctor 2013). Time of origin of the parasitiform lineage is more obscure, and this group is represented by fewer, more-recent fossils (~100 mya; Dunlop and Bernardi 2014, Peñalver et al. 2017).

Reliable identification of any given mite to species is difficult because of the general lack of identification tools, including species-level keys, adequate species descriptions and illustrations, as well as published checklists. This is in stark contrast with major groups of insects and spiders for which there are usually checklists available at the national or provincial levels for the entire order as well as many species-level identification keys. The slow progress in mite systematics reflects the dearth of acarological taxonomists in Canada and North America, the relatively rare inclusion of mites in biological surveys and student projects, and the small number of amateur collectors (Lumley et al. 2013).

While a broad first ‘sweep’ of collecting across Canada was done prior to the year 2000 (Lumley et al. 2013), sampling points are widely scattered with a few exceptions such as Vancouver Island (British Columbia) and Alberta for Oribatida (e.g., Walter et al. 2014, Lindo and Clayton 2017). Labrador is virtually unsampled for mites (Lindquist et al. 1979) and Saskatchewan is poorly explored (Lumley et al. 2013). Similarly, while past collecting efforts were ecologically broad, all habitats including plants and animals (both vertebrate and invertebrate) are in need of targeted exploration for uncovering additional species and clarifying species’ ecological and geographical distributions (Lumley et al. 2013).

Since 1979, several regional assessments have been published for Canada, particularly for soil and freshwater mites of Montane Cordillera (Smith et al. 2011), Mixedwood Plains (Smith et al. 1996), and Atlantic Maritime ecozones (BehanPelletier 2010, Smith 2010); grasslands of western Canada (BehanPelletier and Kanashiro 2010); the Arctic (Behan 1978, Danks 1981) and Yukon (BehanPelletier 1997a); Sable Island (Majka et al. 2007); Cape Breton Highlands National Park (BehanPelletier et al. 1987); and the Churchill, Manitoba region (Young et al. 2012). More restricted ecological surveys have been published for mites in forest canopies of the Pacific coast (Fagan et al. 2006, Lindo and Winchester 2006, 2009), in soil and litter (e.g., St. John et al. 2002, Déchêne and Buddle 2009, BehanPelletier and Kanashiro 2010, Sylvain and Buddle 2010, Walter and Latonas 2012, Newton 2013, Meehan and Turnbull 2018), peatlands (BehanPelletier and Bissett 1994, Barreto and Lindo 2018, McAdams et al. 2018), in dung (Lindquist 1998), and on plants (Forest et al. 1982, Beaulieu and Knee 2014), bumble bees (Haas et al. in press), beetles (Lindquist and Wu 1991, Knee et al. 2013), and birds (Galloway et al. 2014, Knee and Galloway 2017b). For certain families or higher taxa of Acari, these publications represent a key source of information for species records in Canada.

## Methods

Described species records in Canada were compiled based on a critical assessment of the literature as well as unpublished specimen records from collections, primarily the Canadian National Collection of Insects, Arachnids and Nematodes (CNC), but also from the following: the Royal Alberta Museum (PMAE), JB Wallis/RE Roughley Museum of Entomology at the University of Manitoba, the Zoological Institute in St. Petersburg, Russia, H Proctor’s collection (University of Alberta), Z Lindo’s collection (Western University), the Habeeb Collection (New Brunswick Museum), the Ohio State University Acarological (Laboratory) Collection (OSAL; online database: available from https://acarology.osu.edu/database), the United States National Museum (USNM), and the University of Michigan Museum of Zoology (UMMZ). Note the difference between the general approach of Lindquist et al. (1979) and our approach for assessing the number of known species. Lindquist et al. (1979) provided an estimate of the number of species represented by specimens in hand (in addition to the rare published records), including the relatively few named species and variously identified morphospecies. In contrast, in this treatment ‘known species’ includes only named species. This explains some of the apparent decreases in the ‘no. known spp.’ between 1979 and current numbers for some families in Tables 2–6; other reductions are due to changes in family classifications of genera.

We have excluded mites that survive only on introduced hosts that do not occur naturally outdoors in Canada, or cannot become naturalized because of intolerance to our climate. This excludes numerous host-specific mites associated with exotic zoo animals, the pet trade (e.g., tropical birds and lizards), and with cultures of tropical insects. However, we have included mites that are regularly encountered in greenhouses or in stored products, even if some may not survive Canadian winters, because these species are more tractable and are from temperature-protected major ‘agricultural environments’.

While estimates of unrecorded and undescribed species for each family are fairly subjective, they are often based on concrete information: (1) Canadian specimens determined to be new, undescribed species; (2) Canadian specimens not yet determined to species, or only tentatively, but that most likely represent a number of additional distinct species; (3) published or unpublished collection-based records (particularly from OSAL, USNM, UMMZ) of species from northern USA with ranges in close proximity to Canada, or from the USA and associated with host species that also occur in Canada. We also considered the habitats and hosts that are unexplored for particular mite groups and the level of host specificity for symbiotic mites, when available. In addition, we adjusted our estimates with the magnitude of faunal records for families that are better known in other regions of the world with a similar climate, e.g., European countries (Karg 1989, 1993, Biesiadka et al. 1997). Molecular data (BINs: Barcode Index Numbers) were used minimally to estimate unrecorded species, except when supported by morphological data that suggest delineation of undescribed species.

The information sources presented in Tables 2–6 are not exhaustive but are the most useful publications that include new records, species lists or taxonomic revisions. When a publication refers to many families of a given superfamily, it is listed for the superfamily. Additional relevant sources are presented in the main text. Note that collections, particularly the CNC, are an information source of unpublished species records for most families presented, but for the sake of simplicity were not indicated as sources in Tables 2–6.

Family, superfamily and higher-level classification mainly follow Lindquist et al. (2009a), as modified by Beaulieu et al. (2011) for Parasitiformes, Zhang et al. (2011) for Trombidiformes, Walter et al. (2011) for Endeostigmata, and Schatz et al. (2011) for Oribatida. Further modifications were made based on more recent publications which are cited in the text or table footnotes. Names of the hyporders of Mesostigmata do not follow Beaulieu et al. (2011), but rather more traditional publications (e.g., Epicriina instead of Epicriae; see the endnotes in Lindquist et al. (2009a) for sources), similar to what Zhang et al. (2011) and Schatz et al. (2011) did for Trombidiformes and Oribatida.

The text below is generally divided into superfamilies except in cases where a broader, more inclusive treatment (e.g., Oribatida excluding Astigmata) was deemed more practical, for instance, because the ecology of the entire group is relatively homogeneous.

Because current species records are so patchy, determining the range of occupied ecozones for a given family is difficult and often required subjective extrapolation of the family’s distribution across ecozones. For families of vertebrate symbionts (ticks and many Astigmata, Prostigmata, and Mesostigmata), ecozones occupied were often in part inferred from the known distribution of the host groups rather than from the typically limited collection records for the mites themselves. For those families, the host group was indicated in the ecozone column in order to be more informative. Note that an association to a vertebrate host can be parasitic, commensal (e.g., putatively most cases of phoresy), or even mutualistic. Extrapolation was also done for families which are not yet recorded in Canada, by inference from northern USA records and/or host ranges. Distribution across ecozones indicated in tables should therefore be interpreted with caution.

## Overview of faunal diversity

There are 2999 named species from 269 families currently recorded in Canada, including at least 1082 new records since 1979, a 56% increase (Table 1). This increase would be even more substantial, if there were not a discrepancy in the method of reporting ‘recorded species’ in 1979 vs the present treatment (see Methods). An additional 42 families are represented only by material determined to family- or genus-level (these families have no known species included in Tables 2–6; see also footnote * under each table). Of the total 311 families known in Canada, as many as 69 are newly recorded since 1979, 27 of which are based on undetermined material only. These new family records exclude apparent new records due solely to classification changes.

Across higher taxa, the number of known species has increased 37–67%, except for the hyporder Astigmata, which has increased 211% (Table 1). This major taxonomic leap for Astigmata is essentially due to the ongoing collaborative research on feather mites, Pterolichoidea and Analgoidea (e.g., Galloway et al. 2014). There is also a major increase in recorded species of Oribatida associated with a series of published checklists and taxonomic revisions (e.g., BehanPelletier and Eamer 2009, BehanPelletier 2013, Walter et al. 2014, Lindo 2018). Among the Trombidiformes, the Hydrachnidia (water mites) is the group with most notable growth in knowledge (e.g., Smith 1992a, Smith et al. 2015), and recent work on Rhinonyssidae represents the most salient progress for Mesostigmata (Knee and Galloway 2017b). Our knowledge of Canadian ticks (Ixodida) benefited from the recent publication of a handbook with diagnoses and keys to all established species in Canada (Lindquist et al. 2016).

We estimate that over 6600 species of mites are as yet unrecorded or undescribed in Canada (Table 1). This represents ~70% of the total expected fauna. Our total estimate (known plus unknown species) of 9628 species is close to the ~9500 of Lindquist et al. (1979). We consider our estimate to be conservative and that as many as 15,000 species could be found in the country.

**Table 1.** Census of Acari in Canada.

Taxon	No. species reported in Lindquist et al. (1979)^1^	No. species currently known from Canada	No. BINs^2^ available for Canadian species	Est. no. undescribed or unrecorded species in Canada	No. families in Canada			
					with ≥1 named species	with undet. species only	with BINs	anticipated (not yet recorded)
**Superorder Parasitiformes**								
Order Ixodida	33	48	34	10	2	0	1	0
Order Mesostigmata	473	650	1409	967	46	8	36	4
**Superorder Acariformes**								
Order Trombidiformes**^3^**	802	1100	3261	2162	86	14	62	10
Order Sarcoptiformes								
Suborder Endeostigmata**^3^**	113	168	176	1049	8	2	≥4	2
Suborder Oribatida**^4^**	354	592	2429	1267	84	15	67	1
Hyporder Astigmata	142	441	153	1174	43	3	19	12
**Total**	**1917**	**2999**	**7462**	**6629**	**269**	**42**	**189**	**29**

**^1^**In 1979, the two column headings for species counts were “Estimated no. Can. species rec. (1) and uncoll. (2)”; the term “rec.” included named species as well as, in many cases, species that were not assigned to species names but that represented distinct, and potentially undescribed, species; the term “uncoll.” included potential species that were not represented by specimens. In contrast, for our current assessment, “No. spp. currently known” includes only named species (see Methods). **^2^**Barcode Index Number, as defined in Ratnasingham and Hebert (2013). **^3^**Lindquist et al. (1979) reported 913 species of ‘Prostigmata’, which included families now in Endeostigmata, as well as Eriophyoidea, also currently included in Endeostigmata, not in Trombidiformes (following Bolton et al. 2017, Klimov et al. 2018); furthermore, two additional species (of Linotetranidae and Camerobiidae) were recorded only in footnotes in Lindquist et al. (1979) and not included in the total of 913. **^4^**Values exclude Astigmata.

### Molecular data for species delineation and diversity assessment

Since Lindquist et al. (1979), many molecular markers (e.g., ITS, COI, 16S, 18S, 28S) have been used to help elucidate species boundaries in the Acari (Navajas and Fenton 2000, Cruickshank 2002, Navajas and Navia 2010, dos Santos and Tixier 2017, Lehmitz and Decker 2017). In particular, the 658 bp ‘barcode’ region of COI (cytochrome *c* oxidase subunit I) has been promoted as a reliable determinant of species boundaries in animals (Hebert et al. 2003); however, compared to other arthropod groups (Virgilio et al. 2010, Wilson et al. 2017), the paucity of barcode data available for Acari worldwide is striking (e.g., Navajas and Navia 2010). This also applies to Canada, where 70–80% of all named Canadian Lepidoptera, Coleoptera and Araneae have been barcoded and assigned BINs, with each BIN representing a ‘barcode cluster’ for a putative species (Ratnasingham and Hebert 2013, Blagoev et al. 2015, Brunke et al. 2019, Pohl et al. 2019), whereas fewer than 10% of named Acari have assigned BINs. Nevertheless, 7462 BINs from 189 families have been sequenced for Canadian Acari (Tables 1–6; available in BOLD). Interestingly, the BIN richness is well above past and present predictions of species richness for many families in Canada (see further notes in respective taxonomic sections below; Lindquist et al. 1979). Note also that available BINs are based on a limited number of samples, habitats and hosts surveyed. For instance, ~900 of these BINs were generated from a single barcode study of mites at one location in subarctic Canada (Young et al. 2012).

Because of variation in mutation rates among mite taxa, variable retention of ancestral polymorphisms, and past hybridization events, the BIN algorithm may overestimate species richness in some groups. High intraspecific divergences in mites (7–20%) have been observed in some cases (e.g., Heethoff et al. 2007, Niedbała and Dabert 2013, Rosenberger et al. 2013, Zhang and Zhang 2014), possibly reflecting higher rates of mitochondrial evolution in mites than in most other animal taxa (Young and Hebert 2015), or long-term consequences of reproductive systems such as haplodiploidy (Tixier et al. 2017) and thelytoky (Palmer and Norton 1992). On the other hand, it is plausible that cases of high BIN richness, at least in part, reflect cryptic diversity that is not yet resolved morphologically (e.g., Ratnasingham and Hebert 2013, Ondrejicka et al. 2016). Indeed, species distinguished by only minor morphological differences, or cryptic species, are common and sometimes associated with narrower host ranges than previously thought (Knee et al. 2012a, Miller et al. 2013).

The barcode region has been useful for delineating species in various groups of parasitiform and acariform mites (e.g., Roy et al. 2009, Knee et al. 2012a, Glowska et al. 2014, Doña et al. 2015, Ondrejicka et al. 2016, Fisher et al. 2017). In Canada, approximately 60–70% of 230 morphologically recognized species of oribatids across 45 families are currently supported by barcode clusters (M Young and L Lumley unpubl. data). The rapidly growing barcode reference library for the Acari of Canada is therefore of considerable use. However, the most important and possibly most onerous step, the association of DNA sequences with morphological concepts, has to be addressed for most taxa in order to test the effectiveness of barcode-based species identifications. To strengthen analyses, a multigene approach should be favoured in the future, especially given the fast development of next-generation sequencing technology (Marcus 2018). With such increasing ease in obtaining sequences, it is more critical now than ever to build morphologically verified voucher libraries that will enable sequencing results to be taxonomically and ecologically meaningful.

### Adventive species

Like almost all biota of this previously largely ice-covered country, the acarofauna of Canada has been recovering from glaciation since ice sheets began retreating ~14,000 years ago (Menounos et al. 2017). Consequently, determining which species have been introduced through recent human activity is often impossible, complicated by a lack of geographic, taxonomic, phylogenetic or host data (Langor et al. 2009, Beaulieu and Knee 2014). Even cases of mites specific to animal or plant hosts that have been introduced may present difficulties, because of uncertainties in the mite species boundaries, the actual host range, or biogeographical history of the host (Mironov et al. 2005, Navajas et al. 2010, Miller et al. 2013). Undoubtedly, many mite species have been inadvertently introduced to Canada with soil or water ballasts emptied near ports; with vertebrate hosts introduced intentionally as livestock, as pets or for hunting, or accidentally with imported goods, e.g., rodents (Navajas et al. 2010); and with invertebrate hosts on which they are parasitic or phoretic, such as fly pests, wood-boring beetles, or dung beetles introduced to accelerate decomposition of cattle dung (Humble and Allen 2006, Floate 2011). Widespread agricultural pests, such as the two-spotted spider mite, and many of the cosmopolitan mites breeding in stored grains, insect or fungal cultures, or bee hives (e.g., *Varroa*, *Acarapis* spp.), clearly originate from outside Canada (Langor et al. 2009, Beaulieu and Knee 2014). A few species have been intentionally introduced for biocontrol of agricultural pests or weeds (McClay and De Clerck-Floate 2013). In Europe, 96 species of mites have so far been identified as adventive, most of which are associated with agricultural commodities (Navajas et al. 2010). Given international plant trade, and the small size, cryptic habits and dispersal potential (e.g., phoresy, wind) of mites (Navajas et al. 2010, Beaulieu and Knee 2014, NAPPO 2014), we can expect a considerable number of non-native mites to be present already in Canada, and that their numbers will increase.

## Superorder Parasitiformes

Parasitiform mites represent one of the two currently recognized and apparently phylogenetically distant acarine lineages. Only two of the four orders are present in Canada, the Ixodida (ticks) and Mesostigmata. The majority of Parasitiformes feed on fluids taken from their prey or hosts, depending on whether they are predators or parasites—the two dominant lifestyles of parasitiforms. A few taxa (e.g., some ameroseiids, ascids, melicharids, phytoseiids, uropodines) feed on pollen, nectar or fungal tissues, including some that ingest particulate matter, and some insect symbionts (‘paraphages’) may feed on secretions of their hosts.

### Order Ixodida

Ticks, the most infamous mites of all, are obligate blood-feeding ectoparasites of terrestrial vertebrates, primarily birds and mammals, but also reptiles and amphibians. Ticks are major threats to wildlife and public health, as they can harm their hosts through exsanguination, paralysis, and transmission of pathogens, including the causative agent of Lyme disease, *Borreliaburgdorferi* (Nicholson et al. 2009, Lindquist et al. 2016). The recent handbook to the ticks of Canada by Lindquist et al. (2016) represents a much-needed update from Gregson (1956) and includes identification keys to all instars as well as information on the species’ life histories, distributions, hosts, and pathogens transmitted.

The known diversity of Ixodidae, or hard ticks, in Canada has increased from 26 in Lindquist et al. (1979) to 41 species (Table 2). However, from this number, approximately 10 *Amblyomma* and five *Ixodes* species represent extralimital records. These species have been reported primarily from migratory birds and, therefore, it is unlikely they have established year-round populations in Canada (Scott and Durden 2015a, b, Lindquist et al. 2016). In the past 20 years, the blacklegged tick, *Ixodesscapularis* Say, a major vector of the agent of Lyme disease, has significantly expanded its range northward from the USA and has become established from Manitoba to Atlantic Canada (Leighton et al. 2012).

Argasidae, soft ticks, are generally nocturnal and more rapid feeders than ixodids, and mainly parasitize bats and birds. The number of known species in Canada has not changed since Lindquist et al. (1979), remaining at seven. However, over half of known argasid species are represented by only one or two records, suggesting that this apparently poor diversity may be in part a result of the lack of study in the country (Lindquist et al. 2016). The absence of BINs generated for Argasidae in Canada supports this hypothesis (Table 2). In contrast, a majority of Ixodidae has been studied molecularly, with at least 26 of the 34 BINs generated assigned to named species (Ondrejicka et al. 2016). It is expected that a few additional species of ticks in both families will eventually be found or recognized in Canada (Table 2).

**Table 2.** Census of the superorder Parasitiformes (Acari) in Canada.

Taxon^1^	No. species reported in Lindquist et al. (1979)	No. species currently known from Canada	No. BINs^2^ available for Canadian species	Est. no. undescribed or unrecorded species in Canada	General distribution by ecozone^2A^ and vertebrate host range	Information sources
**Order Ixodida**						
**Superfamily Ixodoidea**						Lindquist et al 2016
Argasidae	7	7	0	2	Boreal ecozones and southward; birds, bats, other mammals	
Ixodidae	26^3^	41	34	8	all ecozones; mammals, birds	Guglielmone et al. 2004
**Order Mesostigmata**						
**Suborder Sejida**						
**Superfamily Sejoidea**						
Ichthyostomatogasteridae*	1^4^	0	0	1	Mixedwood Plains	
Sejidae	1	3	3	5	Taiga ecozones and southward	
Uropodellidae	?^4^	0	0	1	Mixedwood Plains	
**Superfamily Heterozerconoidea**						
Heterozerconidae*	0	0	0	1	Mixedwood Plains	Gerdeman and Klompen 2003
**Suborder Trigynaspida**						
**Infraorder Cercomegistina**						
**Superfamily Cercomegistoidea**						
Cercomegistidae	0	0	1	1	Pacific Maritime, Mixedwood Plains	Kinn 1970
**Infraorder Antennophorina**						
**Superfamily Antennophoroidea**						
Antennophoridae	0	1	0	3	Pacific Maritime, Prairies (Cypress Hills), Mixedwood Plains	Wheeler 1910, Wisniewski and Hirschmann 1992
**Superfamily Celaenopsoidea**						
Diplogyniidae	4	1	1	4	Mixedwood Plains	
Euzerconidae	1	1	0	0	Mixedwood Plains	Funk 1980
**Superfamily Paramegistoidea**						
Paramegistidae	0	0	0	1	Mixedwood Plains	Nickel and Elzinga 1970
**Superfamily Parantennuloidea**						
Parantennulidae	1	1	0	1	Mixedwood Plains	
Philodanidae	1	1	0	0	Mixedwood Plains	Kethley 1977
**Suborder Monogynaspida**						
**Infraorder Uropodina**						Halliday 2015, 2016
**Superfamily Microgynioidea**						
Microgyniidae	1	1	7	3	Montane Cordillera, Western Interior Basin, Boreal Shield	Meehan and Turnbull 2018
**Superfamily Thinozerconoidea**						
Protodinychidae	1	1	0	0	Mixedwood Plains, Boreal Shield	Hutu and Calugar 2002
**Superfamily Uropodoidea**						
Cillibidae	0	0	0	2	Mixedwood Plains	
Dinychidae	?^5^	3	19	15	all ecozones	
Discourellidae	?^5^	5	0	5	Pacific and Atlantic Maritime, Mixedwood Plains	Hiramatsu and Hirschmann 1979
Metagynuridae	0	0	0	2	Mixedwood Plains	
Oplitidae	0	0	2	10	Prairies, Boreal Shield, Atlantic Maritime	
Polyaspididae	15^6^	0	6	10	most ecozones	
Trachytidae	>2^6^	10	14	25	Montane Cordillera, Taiga Plains, Mixedwood Plains, Atlantic Maritime	Hutu 2000
Trachyuropodidae	1	1	1	20	Prairies, Mixedwood Plains, Atlantic Maritime	Kontschan et al. 2010
Trematuridae **^7^**	?**^5^**	30	31	40	Boreal ecozones and southward	Hirschmann 1978, Hirschmann and Wisniewski 1987, Knee et al. 2012a, c, 2013
Urodiaspididae	?**^5^**	2	0	5	Pacific and Atlantic Maritime	
Urodinychidae **^8^**	?**^5^**	10	9	35	Boreal ecozones and southward	Hirschmann 1979, Knee et al. 2012a, b
Uropodidae	29**^5^**	3	5	25	Mixedwood Plains, Atlantic Maritime	Majka et al. 2007
**Superfamily Diarthrophalloidea**						
Diarthrophallidae	1	1	0	0	Mixedwood Plains	Schuster and Summers 1978
**Infraorder Gamasina**						
**Hyporder Epicriina**						
**Superfamily Epicrioidea**						
Epicriidae	2	5	1	5	Boreal ecozones and southward	Moraza and Johnston 2004, Moraza 2005
**Superfamily Zerconoidea**						
Coprozerconidae*	0	0	0	1	Mixedwood Plains; woodrat nests	Moraza and Lindquist 1998
Zerconidae	30	41	62	50	all ecozones	Diaz-Aguilar and Ujvari 2010, Sikora 2014
**Hyporder Arctacarina**						
**Superfamily Arctacaroidea**						
Arctacaridae	2	2	3	5	Boreal ecozones and northward	Makarova 2003
**Hyporder Parasitina**						
**Superfamily Parasitoidea**						
Parasitidae	35	51	123	100	all ecozones	Richards and Richards 1976, Poprawski and Yule 1992, Walter and Latonas 2012
**Hyporder Dermanyssina**						
**Superfamily Veigaioidea**						
Veigaiidae	10	9	22	15	all ecozones	Hurlbutt 1984
**Superfamily Rhodacaroidea**						
Digamasellidae	20	39	181	60	Taiga ecozones and southward	Shcherbak 1985, Knee et al. 2012c, 2013
Halolaelapidae	10	2	24	40	all ecozones	
Ologamasidae	10	7	35	20	all ecozones; mammals (phoresy)	Hagele et al. 2005
Rhodacaridae	5	1	9	25	Boreal ecozones and southward	
**Superfamily Eviphidoidea**						
Eviphididae	5	7	8	15	Boreal ecozones and southward	Rigby 1996
Macrochelidae	15	26	31	35	Boreal ecozones and southward; mammals (phoresy)	Krantz 1998, Majka et al. 2007, Knee 2017a
Pachylaelapidae	5	1	20	15	Pacific and Atlantic Maritime, Prairies, Mixedwood Plains	
Parholaspididae	7	4	13	20	Boreal ecozones and southward	Marshall 1964, Marshall and Kevan 1964
**Superfamily Ascoidea**						
Ameroseiidae	10	5	22	20	Boreal ecozones and southward; rodents (phoresy)	Elsen and Whitaker 1985, Mašán 2017
Ascidae	60**^9^**	40	131	40	all ecozones	Beaulieu et al. 2008, Lindquist and Makarova 2011, Makarova and Lindquist 2013
Melicharidae	20**^9^**	26	55	20	all ecozones; rodents, birds (phoresy)	Lindquist and Wu 1991, Lindquist 1995, Karg 2005
**Superfamily Phytoseioidea**						
Blattisociidae	20**^9^**	32	85	25	all ecozones	Lindquist 2003
Otopheidomenidae*	1	0	0	3	Mixedwood Plains	Treat 1975
Phytoseiidae	75	110	320	75	all ecozones	Chant and Hansell 1971, Chant et al. 1974, Denmark and Evans 2011
Podocinidae	0	1	1	0	Mixedwood Plains	Lindquist and Wu 1987
**Superfamily Dermanyssoidea**						
Dermanyssidae	4	11	2	3	all ecozones; birds, mammals	Moss 1978, Knee 2008
Entonyssidae	0	1	0	3	Montane Cordillera; snakes	Fain 1961, Fajfer 2012
Haemogamasidae	9	10	0	10	all ecozones; small mammals	Williams et al. 1978
Halarachnidae	4**^10^**	3	0	5	Taiga ecozones and southward; mammals	Kim et al. 1980
Iphiopsididae	?**^11^**	0	0	5	Boreal Shield, Mixedwood Plains	Farfan and Klompen 2012
Ixodorhynchidae	5	3	0	2	Mixedwood Plains; snakes	Johnston 1962
Laelapidae	38**^11^**	61	155	60	all ecozones; small mammals, birds	Crozier 1989, Whitaker et al. 2007
Macronyssidae	7	11	2	20	all ecozones; birds, bats and other small mammals, snakes	Knee and Proctor 2007, Czenze and Broders 2011
Rhinonyssidae	3	61	4	50	all ecozones; birds	Knee 2008, 2018, Knee et al. 2008, Knee and Galloway 2017b
Spinturnicidae	2	4	0	5	Boreal ecozones and southward; bats	Poissant and Broders 2008, Czenze and Broders 2011
Varroidae	0	1	1	0	Boreal ecozones and southward	Currie et al. 2010
**Total**	**506**	**698**	**1443**	**977**		

*These families are anticipated for Canada although no specimens have been collected to date; for other families with 0 known species, some undetermined material has been collected in Canada. **^1^**Classification mostly follows Lindquist et al. (2009b), as slightly modified by Beaulieu et al. (2011), with exceptions mentioned in footnotes 7 and 8. **^2^**Barcode Index Number, as defined in Ratnasingham and Hebert (2013). **^2A^**See figure 1 in Langor (2019) for a map of ecozones. **^3^**Includes former Amblyommidae. **^4^**Probably represents *Uropodella* sp., which is now placed in Uropodellidae. **^5^**Dinychidae, Discourellidae, Trematuridae, Urodiaspididae and Urodinychidae were included in Uropodidae. **^6^**Trachytidae was included in Polyaspididae, and current Trachytidae includes former Dithinozerconidae. **^7^**Includes Nenteriidae (based on H Klompen pers. comm.). **^8^**Includes Uroactiniidae (based on H Klompen pers. comm.). **^9^**Melicharidae and Blattisociidae were included in Ascidae (100 total species reported in 1979), with ~20 species each. **^10^**Includes former Raillietidae. **^11^**Iphiopsididae was included in Laelapidae.

### Order Mesostigmata

This order of mites includes the most diverse group of predatory arthropods in soils and other detrital habitats (e.g., rotting wood, dung, carcasses, nests), a large component of plant-dwelling predators (mostly Phytoseiidae), as well as various lineages of vertebrate parasites (within Dermanyssoidea) and arthropod symbionts including numerous species that disperse phoretically on their hosts. Mesostigmata (= Gamasida) represents the bulk of the Parasitiformes, with 650 species in 46 families currently recorded in Canada, compared to 473 species in 1979, with many more unrecorded (Table 2). An additional eight families are represented in Canada by undetermined species. Taxonomic knowledge has strikingly improved for the bird-parasitic family Rhinonyssidae (Knee and Galloway 2017b) and significantly for Phytoseiidae (Denmark and Evans 2011), Trematuridae, Digamasellidae, Macrochelidae, and Laelapidae, although records for the latter five families are largely based on unpublished CNC specimen data. We anticipate an overall diversity of more than 1600 species of Mesostigmata, of which at least 60% are to be identified.

Molecular work generated 1409 BINs from 36 families of Mesostigmata (Tables 1, 2), most of which are not assigned to named species. These data indicate high genetic diversity that contrasts with our morphology-based assessments for the families Parasitidae (123 BINs), Digamasellidae (181), Ascidae (131), Blattisociidae (85), Phytoseiidae (320), and Laelapidae (155). For instance, the number of BINs for Phytoseiidae is almost twice the total number of known and unrecorded (estimated) species.

#### Suborder Sejida

Sejida is a relatively species-poor but biologically heterogeneous lineage of mites associated with dead wood and litter, mostly as predators (Sejidae, Uropodellidae) or millipede symbionts (Heterozerconoidea) (Walter and Proctor 1998, Lekveishvili and Klompen 2004). Of the two families in Canada, Sejidae has three identified species, and Uropodellidae is known only from one undetermined species (Table 2). A few additional species of Sejidae are expected from Canada. One species of *Asternolaelaps* (Ichthyostomatogasteridae) known from Michigan is expected to occur at least in neighbouring parts of Canada. Similarly, Heterozerconidae may occur here, based on one species recorded from the millipede *Narceusannulatus* Rafinesque in central Ohio (Gerdeman and Klompen 2003); this millipede occurs in southern Canada (Shelley et al. 2006).

#### Suborder Trigynaspida

Trigynaspids are early-derivative Mesostigmata that are mostly restricted to subtropical-tropical regions (Kim 2004, Lindquist et al. 2009b). They are typically arthropod associates, and the few species whose distribution extends into southern Canada are putatively commensals of scolytine, scarab, carabid, tenebrionid, and passalid beetles, as well as ants. They feed as paraphages, cleptoparasites, or as predators of small invertebrates in their beetle host’s tunnels (Kinn 1971, Kim 2004, Seeman 2007). While only a few additional species are anticipated to be found in southern Canada, their biology and distribution remain largely unknown. Within the infraorder Antennophorina, six families are currently recorded in Canada, though Paramegistidae is represented by an undetermined specimen (Table 2). The infraorder Cercomegistina is only known from one undetermined species of Cercomegistidae.

#### Suborder Monogynaspida: Infraorder Uropodina

Monogynaspida is the largest suborder and contains two infraorders, Uropodina and Gamasina, both of which are well-represented in Canada. Uropodines represent a heterogeneous lineage of mites, with four superfamilies in the present treatment. Following evidence from molecular analysis (Klompen et al. 2007), Diarthrophalloidea and Microgynioidea are here included in Uropodina.

##### Superfamilies Microgynioidea, Thinozerconoidea, Diarthrophalloidea

Microgyniidae is a small Holarctic family of mites, with a few representatives in Canada associated with forest litter and dead wood. The superfamily Thinozerconoidea is represented by one species in Canada, *Protodinychusainscoughi* (Hutu and Calugar 2002), collected from beaver lodges and from beetles in the lodges. Diarthrophallidae, intimately associated with passalid beetles (Schuster and Summers 1978), though with poorly understood relationships, is also represented by one species, *Diarthrophallusquercus* (Pearse and Wharton), associated with the only passalid present in Canada, *Odontotaeniusdisjunctus* (Illiger).

##### Superfamily Uropodoidea

Members of this group have strong dorsal and ventral shields with excavations into which the legs and gnathosoma can be withdrawn, giving them the common name ‘tortoise mites’. Their classification is unstable, with poorly resolved generic and family concepts, although important steps to address this confusion have been taken recently (Halliday 2015, 2016). Some of the families presented here (Table 2) may therefore be synonymized in the future. While uropodoid mites thrive in many soil habitats of the southern hemisphere (Beaulieu 2012), in temperate regions, including in Canada, the core of their diversity resides in patchy, transient habitats such as ant hills, dead wood, stored products, compost, carrion, and vertebrate nests. Deutonymphs of these species are phoretic on insects or vertebrates and can be readily collected from these animals. Although poorly known, feeding habits range from predation to omnivory, including fungi or other microbes in their diet (Lindquist et al. 2009b). Most of what we know of uropodoids in Canada involves those associated with beetles and ants. Further sampling of litter and particularly insects as carriers is expected to quadruple the number of species in Canada from 64 named species to an estimated total of 258 (Table 2).

#### Suborder Monogynaspida: Infraorder Gamasina

Among Mesostigmata, the infraorder Gamasina comprises the great majority of species in temperate to polar regions of the world, including Canada. It represents a monophyletic group, although the boundaries of some families and their relationships are still unclear (Klompen et al. 2007, Lindquist et al. 2009b, Dowling and OConnor 2010a).

##### Superfamilies Epicrioidea, Zerconoidea, Arctacaroidea

One family of each of these superfamilies is recorded in Canada. At least eight new species of Zerconidae have been described from Canada since Halašková’s (1977) revision of the family in the Nearctic region, and many more are to be discovered and described (Table 2; Z Ujvari pers. comm.). Two species of Arctacaridae and five of Epicriidae are known from Canada, and a few additional species of each family are anticipated in Canada, especially in the alpine and northern regions for Arctacaridae. It was recently discovered that epicriids are predators that use their elongate forelegs to ensnare collembolan prey (Alberti 2010). At least some zerconid mites show preferences for nematodes (Walter 1988a). The feeding habits of arctacarids are unknown, although predation is suspected, given their strong, serrate chelicerae. Coprozerconidae, currently known only from one species found in *Neotoma* woodrat nests in Kentucky (Moraza and Lindquist 1998), may also occur in Canada (Table 2).

##### Superfamilies Parasitoidea, Veigaioidea

While there is some work done on the diverse fauna of Parasitidae in North America and nearby Greenland (Hennessey and Farrier 1988, 1989, Makarova 2015), and many specimens are identified to species in Canadian collections, the bulk of the taxonomic work remains to be done. This applies as much to the free-living soil predators (members of the subfamily Pergamasinae) as to those that live in patchy nutrient-rich habitats (e.g., animal nests, carcasses, dung) and disperse phoretically as deutonymphs on insects (Parasitinae). Since 1979, only a few additional records have been published (Poprawski and Yule 1992, Walter and Latonas 2012). Currently 51 species are known from Canada and almost twice as many await discovery (Table 2; I Juvara-Bals pers. comm.).

Like many parasitids, veigaiids are fast-moving, aggressive soil-dwelling predators, thriving especially in temperate forests, but also as far north as the Arctic tundra (Danks 1981, Hurlbutt 1984, Lindquist et al. 2009b). There are nine species known from Canada and at least another 15 expected.

##### Superfamily Rhodacaroidea

The increase of known Digamasellidae in Canada from 20 in 1979 to the current total of 39 species (Table 2) is due more to ecological surveys than to taxonomic revisions. Examination of insects, especially beetles, for phoretic deutonymphs should reveal an even richer digamasellid fauna, including undescribed species (e.g., Knee et al. 2012c, 2013). Two new species of Ologamasidae were recently described from small mammals (e.g., moles, voles, shrews) in western Canada (Hagele et al. 2005), on which they probably simply disperse, as deutonymphs, to get to and develop in the host’s nest. Other ologamasids are typically free-living predators in forest or grassland soils, although some are phoretic on carabid beetles, and others inhabit littoral sands (CNC, Haq 1965). Rhodacaridae, adapted for moving through interstitial spaces of deeper soils, have not been studied in Canada. As well, there is likely an undocumented diversity of Halolaelapidae from litter, dung, carcasses, tidal debris, and the arthropods on which they disperse (Krantz 2016).

##### Superfamily Eviphidoidea

Except for a few scattered records in the literature (Table 2), this group has been largely unstudied in Canada. Based on studies elsewhere (e.g., Krantz and Whitaker 1988, Masan and Halliday 2010), soil-litter habitats, as well as animal dung and nests, carrion, beach wrack, and coprophilous and necrophagous insects on which eviphidids and macrochelids tend to disperse phoretically, should yield a considerable species diversity in Canadian landscapes. There are 38 species known from four families, and 85 additional species are expected in the country (Table 2).

##### Superfamily Ascoidea

At the time of Lindquist et al. (1979), this superfamily included species now spread across two superfamilies, Ascoidea and Phytoseioidea (Lindquist et al. 2009b, Beaulieu et al. 2011). Ascoidea is now represented by three families in Canada. Ascidae are mostly predators, with 40 species known from Canada and perhaps as many more expected (Table 2). New records include several Arctic species in the aptly-named genus *Arctoseius* (e.g., Lindquist and Makarova 2011) as well as new species phoretic on carabids. Other species are anticipated, from genera (*Anystipalpus*, *Maxinia*) recorded from Eurasia with similar habitats or hosts occurring in Canada (Lindquist and Moraza 2009, Lindquist and Makarova 2012).

Species of Ameroseiidae in Canada are known from bracket fungi, animal nests, compost, stored grain and litter, where they apparently feed on fungi. Since Lindquist et al. (1979), moderate increases in species numbers have been recorded. There are five species recorded and at least 20 more expected (Table 2). So far, genera of flower-associated ameroseiids known from Europe (Karg 1993) and subtropical-tropical regions have not been found in Canada.

Among Melicharidae recorded from Canada, *Proctolaelaps* dominates in species richness and diversity of habits, some having diverged from predation and adapted to feeding on fungi and pollen in associations with beetles and bumble bees. Some recently described *Proctolaelaps* from Canada were collected from rodents (Karg 2005; Table 2). Other melicharid species recorded since Lindquist et al. (1979) include symbionts of *Monochamus* beetles, and a fungivore living in the pore tubes of bracket fungi (Table 2). One or two species of *Rhinoseius* or *Tropicoseius*, which feed on nectar and disperse on hummingbirds (Naskrecki and Colwell 1998), may be present in Canada.

##### Superfamily Phytoseioidea

The ecologically diverse family Blattisociidae includes predators, fungivores, and insect symbionts (Lindquist 1963, Makarova 2004, Lindquist et al. 2009b). Many species of the subfamily Platyseiinae are adapted to subaquatic habitats, including two *Platyseius* species recently described from the west and east coasts of Canada (Lindquist 2003). Blattisociinae are parasitic on arthropods, such as *Blattisociuspatagiorum* Treat, which infests noctuoid moths (Treat 1975). Blattisociids need revision in North America, with 32 species recorded and at least 25 more expected in Canada (Table 2).

Otopheidomenidae are obligate parasites of insects. No species are known from Canada; the species recorded by Lindquist et al. (1979) appears to belong to another family, probably Laelapidae. We anticipate a few species in southern Canada, including one recorded in northeastern USA that lives in the tympanic cavities of noctuoid moths (Treat 1975), and possibly others that parasitize various lygaeid, pyrrhocorid and nabid bugs (Krantz and Khot 1962; Table 2).

Among species-rich families of Mesostigmata worldwide, Phytoseiidae is probably the best known, and this applies to Canada too. This is largely because of their known and potential roles as biocontrol agents of agricultural pests (Gerson et al. 2003). The known diversity of phytoseiids in Canada has significantly increased since 1979 to 110 species (Table 2), thanks to taxonomic revisions (e.g., Chant and Yoshida-Shaul 1984, Denmark and Evans 2011) and newly identified specimens in the CNC, which now includes Don Chant’s collection. Despite this fundamental work, distribution and host records of phytoseiids in North America are scattered. We predict another 75 species may be recorded in Canada in the future.

Lindquist et al. (1979) anticipated that a species of Podocinidae may occur in southern Canada, and this was confirmed by a record from Rondeau Provincial Park (Lindquist and Wu 1987). The same species is expected to occur in south coastal British Columbia.

##### Superfamily Dermanyssoidea

This taxonomically and ecologically diverse group of parasitic and predatory mites includes 11 families and 166 recorded species in Canada (Table 2). The large majority of families consist of strictly blood-feeding parasites found on the skin and in the respiratory tracts of birds, mammals, and reptiles (Radovsky 1994, Lindquist et al. 2009b, Dowling and OConnor 2010b). Some are of medical and veterinary importance. Since 1979, there has been moderate increase in the number of species recorded from bats (Macronyssidae, Whitaker et al. 2007; and Spinturnicidae), birds (Dermanyssidae; Knee 2008), and small mammals (Haemogamasidae), most of which are not published. Most notably, the records for Rhinonyssidae, which inhabit birds’ respiratory tracts, have leaped from three to 61 species in Canada (Table 2), thanks to recent research. One species of Entonyssidae, *Entophionyssushamertoni* (Radford), is known from the respiratory passage of the common garter snake in western Canada (Fain 1961); this record was apparently missed by Lindquist et al. (1979).

Laelapidae includes facultative and obligate ectoparasites of mammals (Laelapinae, Hirstionyssinae), species of which are commonly found in their nests. However, the most diverse genera of laelapids are soil-dwelling predators (most Hypoaspidinae) and symbionts of arthropods, particularly ants (other Hypoaspidinae). Species records of Laelapidae in Canada are largely unpublished, and possibly 60 additional species remain to be identified or described in the country (Table 2). A few studies of parasites of rodents and other mammals, of bumble bee associates, and soil surveys have increased records of laelapids in Canada (Jones and Thomas 1982, Whitaker and French 1982, Haas et al. in press). At least three species are sold as biocontrol agents of greenhouse pests, one of which was discovered in Canada (Beaulieu 2009). The large proportion of species identified with uncertainty in Canadian soil surveys (e.g., St. John et al. 2002) is indicative of the need for taxonomic revisions for Laelapidae, especially the free-living Hypoaspidinae.

Although several species of Iphiopsididae are expected to occur in Canada, the only one recorded is an unidentified species of *Narceolaelaps* collected from millipedes. Varroidae is another new family record for Canada since Lindquist et al. (1979). The highly invasive pest of western honey bees, *Varroadestructor* Anderson and Trueman, was recorded in the USA in 1987 and has spread across Canada as of 1989 (Sanford 2001, Rosenkranz et al. 2010).

## Superorder Acariformes: Order Trombidiformes

The superorder Acariformes comprises two lineages, the Trombidiformes and the Sarcoptiformes, which together are far more diverse taxonomically and ecologically than Parasitiformes or any other lineage of arachnids. Like most other arachnids, the vast majority of Trombidiformes feed on fluids (with the possible exception of some Sphaerolichida), but they do so in almost every conceivable way, from predation to mycophagy to plant and animal parasitism. The Trombidiformes consists mostly of the Prostigmata, but includes also Sphaerolichida, which comprises two small families with ambiguous relationships.

### Suborder Sphaerolichida

Sphaerolichidae and Lordalycidae were previously included in the Endeostigmata, in part based on superficial resemblance, but were later transferred to Trombidiformes (OConnor 1984), as they share derived characters with Prostigmata. Molecular work supports this relationship (Pepato and Klimov 2015), and at least Lordalycidae may belong within the traditional Prostigmata (Klimov et al. 2018). These small, globular mites are common in soil, litter and moss worldwide but are rarely studied. The presence of fungal materials in the guts of some of these animals suggests that they are fungivorous (Theron 1979, Walter 1988b). However, it has also been theorized that members of *Sphaerolichus* are ambush predators (Walter et al. 2009). Lordalycids have been collected across Canada, but have not been identified (Table 3). Sphaerolichids, in contrast, are known from just two undetermined specimens from Quebec. A couple more species may be expected for each family.

### Suborder Prostigmata

Prostigmatans display an exceptional diversity of lifestyles and ecological niches, and include herbivores (e.g., Tetranychoidea); fungivores (e.g., many Tydeoidea, Heterostigmata); an array of (proven and putative) predators in soils, on plants, and in fresh and marine waters; arthropod symbionts; diverse lineages of invertebrate (Parasitengona, some Heterostigmata) and vertebrate parasites (e.g., Cheyletoidea, Myobiidae). Based on species records alone, taxonomic knowledge has most notably improved since 1979 for Rhagidiidae, Tydeoidea, Hygrobatoidea, Cheyletoidea, Myobiidae, Stigmaeidae, Tetranychidae, and Tarsonemidae. There are currently 1100 species of Prostigmata recorded in Canada (increased from 800 in 1979), belonging to 86 families (Table 3). This excludes the Eriophyoidea, which, based on recent data (see Bolton et al. 2017, Klimov et al. 2018), appears to belong to Sarcoptiformes. An additional 12 families of Prostigmata are represented in Canada, although by as yet undetermined species, and 10 other families are anticipated. A total of 13 families represent new records since 1979, four of which are based on undetermined material only.

Molecular work yielding barcodes has produced 3261 BINs for 62 families of Trombidiformes (Tables 1, 3). For many families, the number of BINs is well above predictions of total species richness based on morphology, particularly Bdellidae (169 BINs), Cunaxidae (96), Eupodidae (520), Rhagidiidae (165), Tydeidae (217), Anystidae (61), Erythraeidae (313), Trombidioidea (122), and Pygmephoridae (112). We estimate a total diversity of over 3200 species of Prostigmata in Canada, about two thirds of which are yet undocumented.

**Table 3.** Census of the order Trombidiformes (Acari: Acariformes) in Canada.

Taxon^1^	No. species reported in Lindquist et al. (1979)	No. species currently known from Canada	No. BINs^2^ available for Canadian species	Est. no. undescribed or unrecorded species in Canada	General distribution by ecozone^2A^ and vertebrate host range	Information sources
**Suborder Sphaerolichida**						
**Superfamily Lordalycoidea**						
Lordalycidae	2	0	0	2	Taiga ecozones and southward	Marshall and Kevan 1964
**Superfamily Sphaerolichoidea**						
Sphaerolichidae	0	0	0	2	Mixedwood Plains	
**Suborder Prostigmata**						
**Infraorder Labidostommatina**						
**Superfamily Labidostommatoidea**						
Labidostommatidae ^3^	1	1	0	3	Pacific Maritime, Boreal Cordillera, Arctic, Mixedwood Plains	Bertrand 1990
**Infraorder Eupodina**						
**Superfamily Bdelloidea**						
Bdellidae	15	20	169	25	all ecozones	Hernandes et al. 2016
Cunaxidae	10	16	96	40	all ecozones	Skvarla et al. 2014
**Superfamily Eupodoidea**						
Eupodidae	35	13	520	30	all ecozones	Behan 1978, Moseley 2007
Penthaleidae	1	2	13	4	most ecozones	Behan 1978
Penthalodidae	2	2	21	5	all ecozones	Jesionowska 2010
Rhagidiidae	25^4^	51	165	30	all ecozones	Behan 1978, Zacharda 1986, 1995a, b, 1996, 1997
Strandtmanniidae	?^4^	0	0	1	Prairies, Atlantic Maritime	Zacharda 1979, Newton 2013
**Superfamily Tydeoidea**						
Ereynetidae	7	9	23	40	all ecozones; birds	Knee et al. 2008, Knee and Galloway 2017b
Iolinidae	?^5^	12	3	20	all ecozones	Marshall 1970, Forest et al. 1982, Momen 1990, Momen and Sinha 1991, Silva et al. 2016
Triophtydeidae	?^5^	2	18	5	Boreal ecozones and southward	
Tydeidae	20^5^	30	217^6^	40	all ecozones	
**Infraorder Anystina**						
**Hyporder Anystae**						
**Superfamily Adamystoidea**						
Adamystidae	1	0	0	1	Montane Cordillera, Western Interior Basin, Boreal Shield	Ueckermann 1989
**Superfamily Anystoidea**						
Anystidae	2^7^	1	61	3	Boreal ecozones and southward	
Erythracaridae	?^7^	0	8	6	most ecozones	Otto 1999a, b, c
Pseudocheylidae	1	2	0	1	Mixedwood Plains	Van Dis and Ueckermann 1991
Teneriffiidae*	0	0	0	1	Montane Cordillera, Western Interior Basin	Walter et al. 2009
**Superfamily Caeculoidea**						
Caeculidae	1	2	2	2	Montane Cordillera, Prairies	McDaniel and Boe 1990
**Superfamily Pomerantzioidea**						
Pomerantziidae	0	2	0	1	Pacific Maritime, Prairies, Mixedwood Plains	Fan and Chen 2005
**Superfamily Paratydeoidea**						
Paratydeidae	1	2	0	3	Pacific and Atlantic Maritime, Montane Cordillera, Prairies, Mixedwood Plains	Khaustov 2017
**Unplaced superfamily**						
**Superfamily Halacaroidea^8^**						
Halacaridae	10	10	1	100	most ecozones	Bartsch 2011, Chatterjee et al. 2011
**Hyporder Parasitengona**						
**Parvorder Erythraeina**						Mąkol and Wohltmann 2012
**Superfamily Calyptostomatoidea**						
Calyptostomatidae	1	1	13	2	Boreal ecozones and southward	
**Superfamily Erythraeoidea**						
Erythraeidae	10	5	313	50	all ecozones	Southcott 1992b
Smarididae	1	0	14	5	Prairies (Cypress Hills), Mixedwood Plains, Atlantic Maritime	
**Parvorder Trombidiina**						Mąkol and Wohltmann 2012
**Superfamily Tanaupodoidea**						
Amphotrombiidae	0	0	0	2	Pacific Maritime, Boreal Shield	
Tanaupodidae	0	0	0	5	Atlantic Maritime	
**Superfamily Trombiculoidea**						
Johnstonianidae	3	1	22	8	most ecozones	
Leeuwenhoekiidae	?^9^	1	0	15	Boreal ecozones and southward; bats, rodents, amphibians	Walters et al. 2011
Neotrombidiidae*	0	0	0	4	Mixedwood Plains, Atlantic Maritime	Lindquist and Vercammen-Grandjean 1971
Trombellidae*	0	0	0	1	Mixedwood Plains	Southcott 1987
Trombiculidae	7^9^	20	9	70	Taiga Cordillera and southward; mammals incl. bats, birds, herps	Brennan and Jones 1955, Wrenn 1987, Walters et al. 2011
Walchiidae*	0	0	0	1	Boreal Shield, Mixedwood Plains, Atlantic Maritime; rodents, opossum	
**Superfamily Trombidioidea**						
Microtrombidiidae	?^10^	7	80	20	all ecozones	Southcott 1991, 1992a, 1993
Trombidiidae	10^10^	3	42	15	all ecozones	
**Parvorder Hydrachnidia^11^**						Smith 1987b, 1991c, 2010, Smith et al. 2011
**Superfamily Arrenuroidea**						
Acalyptonotidae	2	3	1	2	most ecozones	Smith 1983e
Arrenuridae	100	97	137	125	all ecozones	
Athienemanniidae	5	6	0	1	Taiga ecozones and southward	Smith 1989a, 1990a, 1992a
Chappuisididae ^12^	1	5	0	1	Taiga ecozones and southward	Smith 1983b, 1992b
Krendowskiidae	3	3	3	3	Mixedwood Plains, Atlantic Maritime	Smith 1983a
Laversiidae	1	1	1	1	Taiga ecozones and southward	
Mideidae	3	2	2	1	most ecozones	
Mideopsidae	12^13^	16	22	15	Taiga ecozones and southward	
Momoniidae	3	9	1	3	Taiga ecozones and southward	Smith 1989b, c, d, 1991b
Neoacaridae	3	9	0	5	Taiga ecozones and southward	Smith 1983d, 2003
Nudomideopsidae	?^13^	2	1	0	Taiga ecozones and southward	Smith 1990b
**Superfamily Eylaoidea**						
Eylaidae	6	9	23	20	all ecozones	Smith 1986
Limnocharidae	3	5	6	2	Taiga ecozones and southward	Smith and Cook 2005a, b
Piersigiidae	1	1	1	2	Boreal Shield, Taiga Shield, Mixedwood Plains, Atlantic Maritime	
**Superfamily Hydrachnoidea**						
Hydrachnidae	15	23	21	20	Taiga ecozones and southward	Conroy and McKillop 1984, Smith 1987a
**Superfamily Hydrovolzioidea**						
Hydrovolziidae	3	2	0	1	Boreal ecozones and southward	
**Superfamily Hydryphantoidea**						
Hydrodromidae	2	1	12	4	Taiga ecozones and southward	
Hydryphantidae	30	42	82	20	Taiga ecozones and southward	Smith 1983c, Smith and Cook 1998, 1999a, b, 2009b
Rhynchohydracaridae	0	1	0	2	Pacific and Atlantic Maritime, Mixedwood Plains	
Thermacaridae	0	1	1	0	Montane Cordillera	Heron and Sheffield 2016
**Superfamily Hygrobatoidea**						
Aturidae	30	40	25	40	Taiga ecozones and southward	Smith 1984a, Radwell and Smith 2012
Feltriidae	15	35	3	35	all ecozones	
Frontipodopsidae	0	1	0	0	Pacific Maritime, Montane Cordillera	
Hygrobatidae	20	25	60	60	Taiga ecozones and southward	Conroy 1982a, Conroy and Bilyj 1992, 1998, Smith and Cook 2006
Lethaxonidae	0	1	0	0	Pacific Maritime	
Limnesiidae	15	20	60	40	Taiga ecozones and southward	Smith and Cook 1994
Pionidae	60^14^	65	153	75	all ecozones	Smith 1976, Conroy 1982b, 1984, Smith and Cook 2009a
Unionicolidae	25	35	108	35	Taiga ecozones and southward	Conroy 1991a, b, 1992a, b
Wettinidae	?^14^	2	2	1	Taiga ecozones and southward	
**Superfamily Lebertioidea**						Smith 1982
Anisitsiellidae ^15^	5	10	8	10	Taiga ecozones and southward	Smith 1979b
Lebertiidae	15	20	59	60	all ecozones	
Oxidae	10	9	24	15	Taiga ecozones and southward	
Rutripalpidae	0	1	0	0	Atlantic Maritime	Smith 1991a
Sperchontidae	20	25	28	50	all ecozones	
Teutonidae	2	3	0	3	Taiga ecozones and southward	
Torrenticolidae	15	37	14	25	Taiga ecozones and southward	
**Parvorder Stygothrombiina**						
**Superfamily Stygothrombioidea**						
Stygothrombiidae	?^10^	1	1	10	Taiga ecozones and southward	
**Infraorder Eleutherengona**						
**Hyporder Raphignathina**						
**Superfamily Cheyletoidea**						
Cheyletidae	23^16^	35	9	40	Boreal ecozones and southward; birds, mammals	Thewke and Enns 1979, Bochkov and Galloway 2001
Demodecidae	1	4	0	20	all ecozones; mammals	Gentes et al. 2007, Desch et al. 2010
Harpirhynchidae	2	15	0	40	all ecozones; birds, snakes?	Byers and Proctor 2013, Bochkov et al. 2015
Psorergatidae	4	7	0	40	Boreal Shield, Prairies; mammals	Kok et al. 1971, Giesen et al. 1983
Syringophilidae	0	13	0	300	all ecozones; birds	Skoracki 2011, Zmudzinski and Skoracki 2017
**Superfamily Myobioidea**						
Myobiidae	7	19	0	30	all ecozones; small mammals	Lukoschus et al. 1980, 1988, Whitaker et al. 2007
**Superfamily Pterygosomatoidea**						
Pterygosomatidae*	0	0	0	1	Mixedwood Plains	Newell and Ryckman 1966
**Superfamily Raphignathoidea**						
Barbutiidae	0	1	0	2	Montane Cordillera, Prairies, Mixedwood Plains, Atlantic Maritime	Fan et al. 2003
Caligonellidae	3	4	0	5	Boreal ecozones and southward	
Camerobiidae	1^17^	0	1	6	Pacific and Atlantic Maritime, Montane Cordillera, Western Interior Basin, Prairies	Bolland 1986, 1991
Cryptognathidae	2	2	0	5	Boreal ecozones and southward	Doğan 2008, Walter and Latonas 2012
Dasythyreidae	0	0	0	1	Western Interior Basin, Prairies, Mixedwood Plains	Walter and Gerson 1998, Walter and Latonas 2012
Dytiscacaridae*	0	0	0	1	Boreal ecozones and southward	Mortazavi et al. 2018
Eupalopsellidae	1	1	4	3	most ecozones	
Homocaligidae	2	1	1	2	Boreal ecozones and southward	Fan and Zhang 2004
Raphignathidae	5	4	3	4	Boreal ecozones and southward	McClure 1943
Stigmaeidae	25	40	56	30	all ecozones	Wood 1972, Fan et al. 2016
**Superfamily Tetranychoidea**						Smith et al. 2011, Beaulieu and Knee 2014
Linotetranidae	1^17^	0	0	2	Boreal and Montane Cordilleras, Prairies	Leetham and Milchunas 1985
Tenuipalpidae	5	12	20	40	Taiga ecozones and southward	Beard et al. 2012
Tetranychidae	25	48	154	45	all ecozones	Baker and Tuttle 1994
Tuckerellidae*	0	0	0	1	Prairies	McDaniel et al. 1975
**Hyporder Heterostigmata**						
**Superfamily Dolichocyboidea**						
Crotalomorphidae*	0	0	0	1	Mixedwood Plains	Lindquist and Krantz 2002, Husband and Husband 2005
Dolichocybidae	1	0	0	5	Prairies, Mixedwood Plains	Magowski and Moser 1993
**Superfamily Heterocheyloidea**						
Heterocheylidae	1	1	0	0	Mixedwood Plains	Lindquist and Kethley 1975
**Superfamily Pyemotoidea**						
Acarophenacidae	2	3	0	5	Boreal ecozones and southward	Cross and Moser 1971, Walter and Seeman 2017
Caraboacaridae	0	0	0	2	Mixedwood Plains	Nickel and Elzinga 1969
Pyemotidae	3^18^	6	0	5	Boreal ecozones and southward	Moser et al. 1987
**Superfamily Pygmephoroidea**						
Microdispidae	2	0	3	10	Boreal ecozones and southward	Mahunka and Philips 1978, Smiley and Moser 1984, Uppstrom 2010
Neopygmephoridae	?^18^	9	0	25	all ecozones	Mahunka 1975, Whitaker et al. 2007
Pygmephoridae	25^18^	21	112	50	all ecozones; mammals (phoresy)	Mahunka 1975, Smiley and Whitaker 1984, Whitaker et al. 2007
Scutacaridae	25	6	145	90	all ecozones	Delfinado and Baker 1976, 1978, Ebermann and Jagersbacher-Baumann 2015
**Superfamily Tarsocheyloidea**						
Tarsocheylidae	4	4	5	4	Mixedwood Plains, Boreal Shield, Atlantic Maritime	Delfinado and Baker 1974
**Superfamily Tarsonemoidea**						
Podapolipidae	1	7	0	20	all ecozones	Husband 1998, Husband and Husband 2004, 2007
Tarsonemidae	40	53	79	70	all ecozones	Lindquist 1969a, b, 1970, 1986
**Superfamily Trochometridioidea**						
Trochometridiidae	0	1	0	1	Western Interior Basin, Prairies	Cross and Bohart 1979, Lindquist 1985
**Unplaced superfamily**						
**Superfamily Cloacaroidea**						Bochkov and OConnor 2008
Cloacaridae*	0	0	0	4	most ecozones; turtles, birds	
Epimyodicidae*	0	0	0	2	Taiga ecozones and southward; small mammals	
**Total**	**802**	**1100**	**3261**	**2162**		

*These families are anticipated for Canada although no specimens have been collected to date; for other families with 0 known species, some undetermined material has been collected in Canada. **^1^** Classification mostly follows Walter et al. (2009), as modified by Zhang et al. (2011), with the exceptions mentioned in footnotes 7, 8, 12 and 15, and of Eriophyoidea transferred to Endeostigmata (Table 4), based on Bolton et al. (2017) and Klimov et al. (2018). **^2^** Barcode Index Number, as defined in Ratnasingham and Hebert (2013). **^2A^** See figure 1 in Langor (2019) for a map of ecozones. **^3^** As Nicoletiellidae in Lindquist et al. (1979), see Lindquist and Sidorchuk (2015). **^4^**Strandtmanniidae was included in Rhagidiidae. **^5^**Iolinidae and Triophtydeidae were included in Tydeidae. **^6^** A limited number of BINs may have been misidentified and may represent Triophtydeidae or Iolinidae. **^7^**Erythracaridae was included in Anystidae; Erythracaridae here treated as a separate family following Pepato and Klimov (2015). **^8^** Transferred from Eupodina to Anystina, based on Pepato and Klimov (2015). **^9^**Leeuwenhoekiidae was included in Trombiculidae. **^10^**Microtrombidiidae and Stygothrombiidae were included in Trombidiidae. **^11^** Papers listed here include extensive information and lists of primary references for most or all water mite families that occur in Canada. **^12^** Includes Uchidastygacaridae. **^13^**Nudomideopsidae was included in Mideopsidae. **^14^**Wettinidae was included in Pionidae. **^15^** Includes Bandakiopsidae. **^16^** Includes former Cheyletiellidae. **^17^** Undetermined species of Camerobiidae and Linotetranidae were recorded in Lindquist et al. (1979) in footnotes only, p. 273. **^18^**Neopygmephoridae was included in Pygmephoridae, in part, and Pyemotidae.

#### Infraorder Labidostommatina

The one family in this group, Labidostommatidae, is a distinct, early-derivative assemblage of heavily armoured, predatory mites (Walter et al. 2009). The most recent comprehensive work on labidostommatids is by Bertrand (1990), but no specimens from Canada are mentioned. Based on Canadian records alone, this family appears to be restricted to the west, with specimens from coastal British Columbia, Yukon, and the Northwest Territories; however, as Lindquist et al. (1979) noted, species occurring in the northeastern USA may extend into southern Ontario. One species is presently known from Canada and as many as three more may occur.

#### Infraorder Eupodina

##### Superfamily Bdelloidea

This superfamily includes two families, Cunaxidae and Bdellidae, known as the 'snout mites' due to their elongate gnathosomas. The group is of uncertain monophyly (Pepato and Klimov 2015, Klimov et al. 2018, E Lindquist pers. obs.). Cunaxids and bdellids are predators in soil and litter, but also on plant surfaces (Walter et al. 2009). In their recent catalogue, Hernandes et al. (2016) list only seven species of Bdellidae from Canada. In contrast, Lindquist et al. (1979) included 15 species, and we include 20 recorded species with an additional 25 species expected (Table 3). Similarly, Cunaxidae are represented in Canada by at least 16 described species, with as many as 40 undescribed and/or unrecorded species expected, which is a steep increase since 1979 (M Schwarzfeld and V Nowell unpubl. data; Table 3).

##### Superfamily Eupodoidea

Eupodoid mites live in a range of habitats, including extreme arctic, alpine, coastal and cave environments. Their diet is poorly known but varies from small invertebrate prey (Rhagiididae, perhaps some Eupodidae) to plant tissues (Penthaleidae) and fungi, algae or lichens (suspected in some Penthalodidae and Eupodidae) (Walter et al. 2009). The five known families in Canada include 68 recorded species, which account for roughly half of the expected diversity (Table 3). Within this lineage, Eupodidae and Rhagidiidae are the most species-rich families and are in great need of taxonomic work, as indicated by the number of undescribed species at hand in the CNC and the surprisingly high number of BINs for each. Rhagidiidae was the focus of revisions by Zacharda (Table 3), which included the description of 16 new species from Canada, but clearly much work remains to document this family fully. The putatively predatory family Strandtmanniidae was described by Zacharda (1979); it has been recorded from Alberta (Newton 2013) and New Brunswick, although not identified to named species. While little has changed since 1979 for the depauperate families Penthaleidae and Penthalodidae, the relatively high number of BINs for each suggests additional species remain to be identified.

##### Superfamily Tydeoidea

Tydeoids are small, soft-bodied mites, ecologically diverse, and currently divided into four families (Walter et al. 2009, Zhang et al. 2011), all of which occur in Canada (Table 3). Most appear to be free-living scavengers, predators and omnivores and are common on plant substrates, in soil, moss and lichens. At least 10 species of Iolinidae and Tydeidae have been recorded from stored grains in Canada, including six described as new (Momen 1990, Momen and Sinha 1991). In contrast to the three other families, Ereynetidae also includes vertebrate parasites, six species (subfamily Speleognathinae) of which are recorded as intranasal parasites of birds in Canada (Knee et al. 2008, Knee and Galloway 2017b), and at least another recorded species belongs to a genus (Ereynetinae: *Riccardoella*) of which members are typically blood-feeding parasites of snails and slugs (Table 3). Other newly recorded tydeoids are associated with insects in Canada, including some ereynetids that live beneath tree bark and are phoretic on bark beetles. Also, two genera of iolinids collected from the tympanum of noctuoids in Massachussetts (Treat 1975) probably occur here. In addition to the 53 species of tydeoids known in Canada, we predict that ~100 species will be recorded in the future (Table 3).

#### Infraorder Anystina: Hyporder Anystae

This lineage includes seven families in Canada, five of which are represented by named species (Table 3). Most taxa are considered predatory and inhabit dry, often exposed, habitats (Walter et al. 2009). Pomerantziidae is newly recorded for Canada (unpublished CNC records), with at least two species collected from deep soils. Anystidae, or whirligig mites, is represented by one commonly collected species, *Anystisbaccarum* (L.), thought to be an important biocontrol agent (Gerson et al. 2003). However, molecular data (BINs) suggest that additional anystid species may occur here (Table 3). Erythracaridae, previously treated as a subfamily of Anystidae, was recently elevated to family status (Pepato and Klimov 2015) and is known from Canada based on undetermined material. Known species richness of the families Adamystidae, Pseudocheylidae, Paratydeidae and Caeculidae in Canada has not changed significantly since Lindquist et al. (1979), although one new species of caeculid is being described from Alberta (J Bernard and L Lumley unpubl. data). One additional family, Teneriffiidae, will likely be found on the west coast, as it occurs nearby in the northwestern USA.

#### Infraorder Anystina: Unplaced superfamily

##### Superfamily Halacaroidea

The single family Halacaridae may be more closely related to Parasitengona than to Eupodina (Pepato and Klimov 2015). The majority of Halacaridae known worldwide are from marine littoral or deeper ocean waters (Bartsch 2009); however, half of the 10 recorded species in Canada are from fresh waters, including lakes, rivers and groundwater. It appears the taxonomy of marine taxa is even more poorly known than for freshwater species in Canada, the latter of which were reviewed by Bartsch (2011) for North America. While most halacarids are free-living in sediments or among vegetation as predators or algivores, some are putative parasites as they have been found on or within larger invertebrates, e.g., gastropods, crayfish, and hydrozoans (Walter et al. 2009, Walter and Proctor 2013). As many as 100 additional species are anticipated to be found in freshwater, littoral and marine environments of Canada (Table 3).

#### Infraorder Anystina: Hyporder Parasitengona

Mites of this hyporder have larvae that contrast morphologically and ecologically with the adults. The larvae of most groups are ectoparasitic whereas the deutonymphs and adults are predatory. Parasitengona include many of the largest mites in Canada (2–3 mm) and are often conspicuously coloured, either actively walking on litter, plants or walls (terrestrial parasitengones, including velvet mites), or swimming in freshwater (water mites). Some trombidiids (*Allothrombium*) are biocontrol agents of orchard pests (Zhang 1992).

##### Terrestrial forms (parvorders Erythraeina and Trombidiina)

The adults and deutonymphs of terrestrial Parasitengona mainly inhabit litter, soil and moss throughout Canada where they feed on other arthropods, but a few feed on pollen (Erythraeidae: *Balaustium*; Yoder et al. 2012). Others (e.g., Calyptostomatidae, Johnstonianidae) favour wet habitats near freshwater where hosts (subaquatic Nematocera) occur. Larvae of most families parasitize a wide variety of arthropods whereas those of Trombiculidae and Leeuwenhoekiidae parasitize diverse vertebrate hosts, including humans, and are commonly known as ‘chiggers’.

Families of Erythraeina and Trombidiina have few species known from Canada except Trombiculidae, which represent the only notable increase in recorded species since 1979 (Table 3). Walters et al. (2011) surveyed the literature and provided a summary of known hosts and distributions of chiggers in North America, which includes a few new records from small rodents (Gyorkos and Hilton 1982, Whitaker and French 1982). Based on many undetermined CNC specimens, as well as the numerous records from neighbouring parts of the USA (Walters et al. 2011, Mąkol and Wohltmann 2012), nearly 200 additional species of terrestrial Parasitengona are anticipated for Canada, over half of which are likely to be erythraeids (50 species) and trombiculids (70 species). Since 1979, Tanaupodidae and Amphotrombiidae have been recorded from Canada based on unidentified material from the east coast, and Neotrombidiidae, Trombellidae and Walchiidae are anticipated to be found in Canada (Table 3).

##### Aquatic forms (parvorders Hydrachnidia and Stygothrombiina)

Numerous species in 36 families (Table 3) representing all seven superfamilies of Hydrachnidia, or true water mites, occur in Canada, along with a few species of the enigmatic interstitial family Stygothrombiidae, which is currently classified in a separate monobasic taxon (Walter et al. 2009) and based on molecular data, falls outside of the Hydrachnidia proper (Dabert et al. 2016). Adults and deutonymphs of water mites are active predators of a wide variety of invertebrates in all types of freshwater habitats throughout Canada. Larval water mites are typically parasites of adult aquatic insects but in some cases also utilize immature insects as hosts. Hosts include a variety of insects, especially Diptera, aquatic and semi-aquatic Hemiptera, Odonata and Coleoptera. Stygothrombiidae parasitize Plecoptera.

Knowledge of the identities and distribution of water mite species in Canada has improved substantially since 1979 although much remains to be discovered and published. Comprehensive keys to Nearctic genera have been published (Smith and Cook 1991, Smith et al. 2001, 2010, 2016) and recent improvements in the classification, biology and ecology are summarized in Walter et al. (2009), Smith et al. (2015), and Proctor et al. (2015). Since 1979, several additional families of water mites have been reported from Canada, including Rhynchohydracaridae from streams (Smith 2010, Smith et al. 2011), Thermacaridae from hot springs in the northern Rocky Mountains (Heron and Sheffield 2016), Frontipodopsidae and Lethaxonidae from interstitial waters in western Canada (Cook et al. 2000, Smith et al. 2011), and the first Nearctic record of Rutripalpidae from springs in Nova Scotia (Smith 1991a, 2010). Research is underway on the lebertioid family Torrenticolidae, the hygrobatoid families Feltriidae, Pionidae and Aturidae, as well as the arrenuroid families Mideopsidae, Krendowskiidae, Neoacaridae (*Volsellacarus*) and the extremely diverse genus *Arrenurus* (Arrenuridae). Whereas 568 named species are known to be present in Canada, nearly 700 additional species are expected to be documented in the future (Table 3).

### Infraorder Eleutherengona: Hyporder Raphignathina

#### Superfamily Cheyletoidea

All five families of Cheyletoidea include ectoparasites associated with the skin of their hosts. However, Cheyletidae includes many species that are free-living predators in stored grains, nests, tree bark, bracket fungi, litter, and on insects, especially beetles, e.g., bark beetles, tenebrionids (CNC records; Walter et al. 2009). Other cheyletids are skin parasites of domesticated mammals (e.g., *Cheyletiella*) and of birds, including two new species described from Manitoba (Bochkov and Galloway 2001). Cheyletidae currently comprises 35 known species in Canada, a ~50% increase since 1979, and another 40 species are expected (Table 3).

Canadian records of Demodecidae (four species) are scarce, and include records from hair follicles and sebaceous glands in humans, and from cattle and mule deer (Kennedy and Kralka 1986; Table 3). Certainly many more species occur across the country on mammalian hosts, sometimes causing skin diseases such as demodectic mange and alopecia (Gentes et al. 2007, Walter et al. 2009). The family Psorergatidae, sometimes called itch mites, is represented by seven species in Canada but many more are expected to be found in the skin of mammals (Giesen et al. 1983, Walter et al. 2009).

Harpirhynchidae is another infrequently reported family found on or in the skin of birds, with several new records in Canada since 1979 (Table 3; Bochkov and Galloway 2013). The North American harpirhynchid fauna is poorly known (Bochkov and OConnor 2013), and we expect many more species to be collected in Canada given the diversity of their potential avian hosts. This family may also be discovered infesting snakes in Canada, as in Eurasia (Beron 1974).

Syringophilidae was not recorded from Canada in 1979 but 13 species are now known from the country, and as many as 300 more are anticipated (Table 3; Bochkov and Galloway 2004). Syringophilids live in the quills of feathers, and species tend to be highly host-specific (Skoracki 2011). Unfortunately, they are rarely encountered because collecting these mites usually requires dissection of the quills.

#### Superfamily Myobioidea

The cosmopolitan family Myobiidae comprises blood- and lymph-feeding ectoparasites that cling to the fur of small mammals using forelegs modified for clasping (Bochkov and Fain 2003). Although infrequently collected in Canada, they are known from species of rodents, bats and insectivores that have broad geographic ranges. The known diversity of myobiids in Canada (19 species) has more than doubled since Lindquist et al. (1979), and we expect 30 or more species to be discovered in the future (Table 3).

#### Superfamily Pterygosomatoidea

Pterygosomatidae, the only representative of the superfamily, contains mainly parasites of lizards, but some species parasitize arthropods (Walter et al. 2009). In Canada, they are known only from captive, non-native animals (e.g., chuckwalla: *Sauromalus* sp.) and the family is therefore not considered recorded here. However, one species, *Pimeliaphilussanguisugae* Newell and Ryckman, has been identified from free-living kissing bugs (*Triatomasanguisuga* LeConte) in Michigan, in close proximity to southeastern Canada (Table 3).

#### Superfamily Raphignathoidea

Nine families of raphignathoids are recorded from Canada (Table 3), including two (Barbutiidae, Dasythyreidae) newly recorded since Lindquist et al. (1979), although dasythyreids are known only from unidentified material. While most species are thought to be predatory, some are bryophagous (Lindquist et al. 1979, Walter et al. 2009), including four species of Stigmaeidae described from Canada (Gerson 1972, Wood 1972). The known diversity of the Raphignathoidea in Canada has changed little since Lindquist et al. (1979), except for the arboreal and soil-dwelling family Stigmaeidae, for which the number of known species has increased by over 50%. A few additional species are anticipated to occur for most raphignathoid families, though we expect as many as 30 more for Stigmaeidae (Table 3). Furthermore, a new family of mites, Dytiscacaridae, living as subelytral parasites of dytiscid beetles in the USA (Mortazavi et al. 2018), may later be found in Canada, since some of their host species occur here.

#### Superfamily Tetranychoidea

This is one of the two major groups of Acari that has evolved strict phytophagy, the other being the Eriophyoidea. Three of the five known families are recorded from Canada, with two widespread in the country and representing the bulk of the fauna (Table 3). They include troublesome pests of fruit trees, berries, fields and pastures, greenhouse crops, and ornamentals, as well as potentially invasive species associated with imported commodities, and others that transmit plant viruses (Zhang 2003, Beard et al. 2012, Thistlewood et al. 2013).

The number of known species in this superfamily in Canada has doubled since 1979, and many more species are anticipated (Table 3). Although Tetranychidae, or spider mites, have been revised for the United States (Baker and Tuttle 1994), knowledge of the full North American fauna is limited, with scattered information on the taxonomy, hosts and species distribution (Smith et al. 2011, Beaulieu and Knee 2014). It is thought that approximately half of the Canadian fauna is documented (Table 3).

Tenuipalpidae, commonly called flat mites, is also an understudied group (Gerson 2008, Beard et al. 2012), in part because they are easily overlooked as they are even smaller than spider mites and occupy more cryptic habitats. The fauna of flat mites is known to be considerably more diverse in Canada than was expected in 1979, with many more species to be described, especially in the large genus *Brevipalpus* (Table 3).

Linotetranidae, associated with grass roots (Baker 1953, Leetham and Milchunas 1985), occur in Canada as far north as Yukon, but species are yet to be confirmed (Table 3). The Tuckerellidae, aptly called ‘peacock mites’ for their fan of ornamental setae, are not yet recorded here, but records from South Dakota grasslands suggest they may occur in Canadian prairies (McDaniel et al. 1975, Lindquist et al. 1979).

### Infraorder Eleutherengona: Hyporder Heterostigmata

Heterostigmata comprises seven superfamilies, all represented in Canada, many with highly specialized symbioses with insects (Kaliszewski et al. 1995, Walter et al. 2009).

#### Superfamily Dolichocyboidea

Of the two families constituting this group, the monobasic Crotalomorphidae is a subelytral parasite of carabid beetles recorded from as far north as Michigan and is anticipated to occur in adjacent areas of Canada (Table 3). Dolichocybidae is known in Canada from an undetermined species, and a few additional species are expected. Although closely associated phoretically with insects, dolichocybids seem to be fungivorous, living in habitats conducive to fungal growth (Kaliszewski et al. 1995).

#### Superfamily Heterocheyloidea

Members of the monogeneric family Heterocheylidae evidently live as parasites under the elytra of passalid beetles in decaying wood in widespread areas of temperate and tropical forests, excluding western North America and Europe (Schuster and Lavoipierre 1970, Lindquist and Kethley 1975). As reported previously by Lindquist et al. (1979), only one known species extends with its host into southeastern Canada, and no further species are projected to occur in Canada (Table 3).

#### Superfamily Pyemotoidea

Of the three families in Canada provisionally included in this group, adult female Acarophenacidae (three species) and Pyemotidae (six species) are parasitoids of immature instars of holometabolous terrestrial insects, including various grain-infesting insects and subcortical beetles. A few additional species of these two families are anticipated (Table 3), and a new genus of Pyemotidae remains to be described, based on identification records acquired by Lindquist in 1990–1992 and reported by Barker (1993). Undetermined Caraboacaridae have been collected from litter in Ontario, and we expect more representatives to be collected from harpaline carabid beetles, their usual hosts (Nickel and Elzinga 1969).

#### Superfamily Pygmephoroidea

Following Khaustov (2008), four families are now recognized in this group, all represented widely across Canada, but with many species identified only to family, including all Microdispidae (Table 3). Members of these families occur mostly in soil or galleries of insects, but species of *Pygmephorus* (Pygmephoridae) are specialized nest associates and phoretic on mammals (Whitaker and French 1982). Generally thought to be fungivorous, the feeding habits of nearly all species of these families are unknown. Females of the genus *Siteroptes* (Pygmephoridae) are economically significant as carriers of fungal pathogens of cereals and other grasses. A much larger variety of taxa of these families is anticipated to occur in Canada.

#### Superfamily Tarsocheyloidea

The one family and two genera of free-living predators that constitute this cosmopolitan group are both represented in Canada (four species; Table 3), where they occur in moss, humus and rotting wood and are sometimes associated with passalid beetles (Lindquist 1976). Although there is no change in number of recorded species, they have been more widely recorded across southern parts of Canada since Lindquist et al. (1979).

#### Superfamily Tarsonemoidea

Of the two families constituting this group, all members of Podapolipidae are obligate parasites of insects (Walter et al. 2009). Although some species have been recorded in Canada since Lindquist et al. (1979) bringing the total to seven, as many as 20 additional species in at least four genera are expected to occur in Canada (Husband 1998; Table 3). Tarsonemidae encompasses many genera with disparate feeding habits, including fungivores, phytophages, parasites and parasitoids of insects, and even predators of other tiny mites (Lindquist 1986). There are 53 species recorded from Canada (Table 3), a modest increase since Lindquist et al. (1979), including records of three additional genera. As many as 70 more species may occur in Canada, possibly including representatives of a newly described tribe living as parasites on tetrigid grasshoppers (Seeman et al. 2018).

#### Superfamily Trochometridioidea

The only family in Canada (Trochometridiidae) was not recorded at the time of Lindquist et al. (1979). First noted by Lindquist (1985), the record of *Trochometridium* from a halictid bee in Penticton, British Columbia, remains the only record for Canada. These associates of ground-nesting bees (Cross and Bohart 1979) are anticipated to occur in arid southern areas of British Columbia and Alberta (Table 3).

### Infraorder Eleutherengona: Unplaced superfamily

#### Superfamily Cloacaroidea

This superfamily consists of specialized parasites of the subcutaneous and mucosal tissues of turtles, birds and small mammals. Some species appear to use the cloacal region of their hosts to disperse between hosts during mating (Bochkov and OConnor 2008). Neither Cloacaridae nor Epimyodicidae have been recorded from Canada, although four species of cloacarids and two species of epimyodicids have hosts that occur in the country.

## Superorder Acariformes: Order Sarcoptiformes

This order unites the large suborder Oribatida, which also includes the Astigmata, with the suborder Endeostigmata. Sarcoptiform mites are unusual among arachnids in that basal groups are particulate-feeders, but fluid-feeding has evolved in several lineages and led to vast radiations on plants (Eriophyoidea) and animal hosts (Astigmata: Psoroptida).

### Suborder Endeostigmata

Endeostigmata represents an early-derivative assemblage of tiny, soft-bodied mites (Walter 2009b). The group is probably not monophyletic and is not well understood phylogenetically, such that its infraorders and superfamilies remain tentative (Pepato and Klimov 2015, Bolton et al. 2017). Since 1979, it has undergone significant classification change, including some family names (Pachygnathidae is now Alycidae), although most constituent genera and families remain the same. The most extraordinary change here is our provisional placement of the Eriophyoidea within Endeostigmata, following recent molecular and morphological evidence that suggests a close relationship between Eriophyoidea and the endeostigmatan family Nematalycidae, both of which consist of worm-like mites that feed on fluids (Bolton et al. 2017, Klimov et al. 2018). In contrast, most sarcoptiform mites swallow particles of food and form discrete gut boluses, although fluid-feeding occurs insome Endeostigmata (e.g., Nanorchestidae, Alycidae: *Bimichaelia*) and several lineages of parasitic Astigmata.

A total of 176 BINs is assigned to at least four families of Endeostigmata (Tables 1, 4), but BINs for Eriophyoidea were not assigned to family level because the families of most specimens need to be verified. Except for Eriophyoidea, which has relatively few BINs compared to known and anticipated species diversity, the other families of Endeostigmata with molecular data (Alycidae, Nanorchestidae, Terpnacaridae) show a contrastingly high number of BINs compared to total expected diversity as inferred from morphological assessment.

#### Endeostigmata in the traditional sense (unplaced families)

Mites of these families are found in soil, litter and moss, but also in extreme conditions such as sand dunes, rocky seashores and High Arctic tundra. From limited information, they feed on fungi, unicellular algae, and/or small invertebrates such as nematodes (Walter 2009b). They are taxonomically poorly known in Canada, with minimal progress since Lindquist et al. (1979) and only 17 species from four families so far recorded (Table 4). Few additional species are expected for Alicorhagiidae and Terpnacaridae, whereas more within the larger families Alycidae and Nanorchestidae may be discovered in the future. Two other families are recorded only from unidentified specimens at hand, and two more are anticipated based on records from nearby USA (Table 4).

**Table 4.** Census of the suborder Endeostigmata (Acari: Acariformes: Sarcoptiformes) in Canada.

**Taxon^1^**	**No. species reported in Lindquist et al. (1979)**	**No. species currently known from Canada**	**No. BINs^2^ available for Canadian species**	**Est. no. undescribed or unrecorded species in Canada**	**General distribution by ecozone^2A^**	**Information sources**
**Unplaced families**						Walter 2009b
Alicorhagiidae	2	3	0	2	Taiga ecozones and southward	Danks 1981
Alycidae ^3^	4	4	24	10	all ecozones	McDaniel 1980, Walter and Latonas 2012
Micropsammidae	0	0	0	1	Prairies	
Nanorchestidae	5	8	86	8	all ecozones	Strandtmann 1982
Nematalycidae*	0	0	0	1	Mixedwood Plains	Bolton et al. 2014
Oehserchestidae	0	0	0	1	Mixedwood Plains	Osler et al. 2008
Proteonematalycidae*	0	0	0	1	Mixedwood Plains	Kethley 1989
Terpnacaridae	2	2	14	5	all ecozones	Walter and Latonas 2012
**Superfamily Eriophyoidea**						Beaulieu and Knee 2014
Diptilomiopidae	10	6	52	75	all ecozones	
Eriophyidae	60	113		900	all ecozones	Marshall et al. 1998, Beaulieu et al. 2016
Nalepellidae ^4^	?^5^	26		20	Taiga ecozones and southward	Marshall and Lindquist 1972, Smith 1984b
Phytoptidae ^4^	30^5^	6		25	all ecozones	Smith 1977
**Total**	**113**	**168**	**176**	**1049**		

*These families are anticipated for Canada although no specimens have been collected to date; for other families with 0 known species, some undetermined material has been collected in Canada. **^1^**Classification is modified from Walter et al. (2011), with the inclusion of Eriophyoidea (previously in Trombidiformes) in Endeostigmata, based on Bolton et al. (2017) and Klimov et al. (2018); and division into infraorders and superfamilies of Walter et al. (2011) is not followed, because of unclear support (Bolton et al. 2017). **^2^**Barcode Index Number, as defined in Ratnasingham and Hebert (2013). **^2A^**See figure 1 in Langor (2019) for a map of ecozones. **^3^**As Pachygnathidae in Lindquist et al. (1979). **^4^**The early-derivative Nalepellidae is herein separated from the angiosperm-associated Phytoptidae, following Chetverikov et al. (2015). **^5^**Nalepellidae was included in Phytoptidae.

#### Superfamily Eriophyoidea

Eriophyoids are often called gall mites or rust mites, referring to the conspicuous galls and deformities induced by many species on their hosts. They are the largest, most ubiquitous group of obligate plant-feeding mites. Their classification is unstable, including family, generic and species concepts, as well as the phylogenetic placement of the superfamily within Acari, which has traditionally been considered a member of Trombidiformes (Lindquist 1996); however, recent data suggest it belongs with the Sarcoptiformes (Klimov et al. 2018). The superfamily is widespread in Canada, likely occurring wherever their hosts occur, which probably includes the large majority of plant species. Some eriophyoids can be especially devastating to agriculture because they vector plant viruses (Oldfield and Proeseler 1996). On the other hand, other species are useful as biocontrol agents of noxious weeds (e.g., Rosenthal 1996, McClay and De Clerck-Floate 2013).

The early-derivative Nalepellidae, herein separated from the angiosperm-associated Phytoptidae (Chetverikov et al. 2015), have diversified on conifer hosts and appear relatively well known in Canada due to a review by Marshall and Lindquist (1972) and to work conducted by Smith (e.g., Smith 1979a, 1984b) (Table 3). Phytoptidae and Diptilomiopidae, each with only six recorded species in Canada, are more poorly known and approximately 25 and 75 species, respectively, are anticipated in the country. Most of the known (113 species) and anticipated diversity (900 species), however, resides in the Eriophyidae. The high proportion of species that could not be identified during a survey of plant-feeding mites in the Prairies of Canada illustrates the need for taxonomic revision of the superfamily (Beaulieu and Knee 2014). Similar to the estimate of Lindquist et al. (1979), we expect over 1000 unrecorded eriophyoid species to occur in Canada based on the large number of potential plant hosts from which mites have not yet been collected, combined with the high host specificity of eriophyoid mites (Oldfield 1996).

### Suborder Oribatida (excl. Astigmata)

Oribatids represent the core lineage of Sarcoptiformes. The numbers presented in this section do not include the hyporder Astigmata, which is taxonomically placed within the infraorder Desmonomata (Norton 1998, Schatz et al. 2011). Due to significant life history differences of the Astigmata compared to the rest of the Oribatida, Astigmata is considered separately (see next section). Oribatid mites inhabit many habitats in addition to their traditional soil-litter environment, such as freshwater (BehanPelletier and Eamer 2007) and marine littoral (Pfingstl 2017), and canopy habitats (BehanPelletier and Winchester 1998, Lindo and Winchester 2007). They are also the dominant arthropods in peatlands (BehanPelletier and Bissett 1994, Barreto and Lindo 2018). Based on their feeding behaviour as a group, they have been referred to as selective generalists (Schneider and Maraun 2005) to reflect the opportunistic feeding strategies of many species within their detrital environment, contributing to decomposition and nutrient cycling processes. Oribatid mites are predominantly detritivore-saprophages, variously feeding on detritus, fungi, algae, and other substrates associated with decaying organic matter, although predation of nematodes is also common. Unique among the Acari and other arthropods is a high prevalence of asexual (thelytokous) reproduction (~9% of species studied), with families or genera for which only females are known (Cianciolo and Norton 2006). Most surprising, however, is the re-evolution of sexual reproductive modes (Domes et al. 2007) arising from within asexual groups, the best example of which is the sexual hyporder Astigmata from within the asexual Desmonomata (Norton 1998). Whether sexual or asexual, many oribatid mites are long-lived for invertebrates (1–7 years; BehanPelletier 1999), with slow development and low reproductive rates (iteroparous).

Despite soil biodiversity in general being relatively understudied (Eisenhauer et al. 2017), the Oribatida have historically been, and remain, one of the better-known groups of soil acarines in Canada, due to concerted sampling efforts. This includes the works of N Banks in the earlier 20^th^ century, as well as some more contemporary sampling efforts by M Hammer (1950s), VG Marshall and colleagues (1960–1970s), M Mitchell (1970s), RA Norton (1970–1980s), and VM Behan/BehanPelletier (1970s–present). Sampling continues to focus on Oribatida, likely due to their charismatic morphology, high diversity, and life-history traits (e.g., K-strategists) that allow them to be included as bioindicators in environmental monitoring. Notable are the extensive works of BehanPelletier and colleagues, including west coast canopy sampling by Z Lindo, and the efforts of DE Walter and colleagues in conjunction with the incorporation of Oribatida into current monitoring programs such as that of the Alberta Biodiversity Monitoring Institute (ABMI) (Walter and Latonas 2012, Walter et al. 2014). Oribatid mites have also often been used for general ecological studies of human and/or natural disturbance (e.g., forestry, agricultural, or industrial practices), and as model taxa for diversity studies (e.g., BehanPelletier 1999, Battigelli et al. 2003, Déchêne and Buddle 2009, McAdams et al. 2018).

Marshall summarized the Oribatida in the Acari section by Lindquist et al. (1979) and reported 71 (+10 expected) families and 354 described species in Canada, with an estimated total diversity of 1554 species. Marshall considered nearly all families (except Kodiakellidae from coastal British Columbia) as probably transcontinental, and the majority of families to have a northern latitudinal range, either to treeline (nine families), to within the Arctic (32 families), or to High Arctic (nine families). The remaining 30 families were considered restricted to southern latitudes. In comparison, of the 100 currently documented or anticipated families in Canada, 44 families are represented in most or all ecozones including the Arctic, 47 families are restricted to boreal and southward, and the remaining nine families are known from only a few ecozones but ranging from southern Canada to as far north as Taiga or Arctic ecozones (Table 5).

Records of Oribatida of Canada up to 1986 were also captured in the *Catalogue of the Oribatid Mites of Canada and the USA* (Marshall et al. 1987), wherein was listed ~300 spp. from 160 genera from published records from Canada. Updates to species and distributional records by province, through to the early 2000s, are available online through the *Diversity of Oribatida in Canada* (DOC) website (BehanPelletier and Eamer 2004).

A total of 592 described species from 84 families are recorded in Canada, a 67% increase since 1979 (Table 5). Another 1267 species are expected to be recorded in the future, approximately 300 of which represent undescribed species or morphospecies currently in collections that need identification or verification. An additional 15 families are known in Canada, based on unidentified specimens, and Lohmanniidae is anticipated to be found. As many as 27 families, including 12 with undetermined species only, represent new records since 1979. The 99 families known in Canada represent 58% of the known world families (currently 172; Norton and BehanPelletier 2009). The families with the highest documented diversity in Canada include (Table 5): Ceratozetidae (53 species), Brachychthoniidae (38), Punctoribatidae (35), Eremaeidae (32), Phthiracaridae (28), Crotoniidae (24), Damaeidae (23), and Oribatellidae (22). Several of these groups have been extensively reviewed by BehanPelletier for North America (Ceratozetidae, Punctoribatidae, Eremaeidae, Damaeidae, and Oribatellidae) and those works contain keys to genera and species (references listed in Table 5).

Families where the number of species has substantially increased since Lindquist et al. (1979) are the Euphthiracaroidea, Crotoniidae, Eremaeidae, Carabodidae, Oribatellidae, and Scheloribatidae (Table 5). Increased species richness in these families is in part due to the taxonomic efforts of VM BehanPelletier, M Colloff, M Reeves, and W Niedbała, concomitant with the exploration of previously unexplored habitats (e.g., canopy habitats). On the other hand, reductions in the number of species in some families (e.g., Suctobelbidae, Oribatulidae) are mostly due to revisions in our understanding of the species concept and/or reassessment and placement of species into other taxonomic groups based on new diagnoses.

Molecular data (BINs) suggest that a large portion of the Canadian oribatid diversity remains undescribed or at least unrecorded, with a total of 2429 BINs recorded across 67 families surveyed so far (Tables 1, 5). However, there are discrepancies between the number of BINs and morphology-based identifications for several families of Oribatida, notably Oppiidae (144 BINs), Tectocepheidae (131), Oribatulidae (175), Scheloribatidae (184), and Ceratozetidae (245). For instance, the low diversity of recorded Tectocepheidae (three known spp.) is in contrast to 131 BINs, despite Tectocepheidae containing only two known genera with a total of 16 described species worldwide (Subías 2004). Within this family, known species in Canada reproduce via thelytokous parthenogenesis, and the number of BINs may indicate high within-lineage diversification (Fontcuberta Garcia-Cuenca et al. 2016). It is interesting to note that while Ceratozetidae has the highest number of described species (53 species) and Punctoribatidae, a closely related family, has similarly high diversity (35 species), the number of BINs recorded for Ceratozetidae is 245 while there are only 35 BINs for Punctoribatidae.

Taking into account the increase in discovered and described species over the last 40 years and the high number of BINs recorded so far, we estimate the diversity of Oribatida in Canada to be at least 1800 species, and possibly as many as 3000 species, with at least two thirds yet unrecorded (Table 5). A comparison of the Canadian oribatid fauna with a recent publication by Krisper et al. (2017) on the Oribatida of Austria, where they document over 677 species, supports the assertion that much of the Canadian fauna is still unknown, given that Canada has ~120 times the area of Austria and contains many more ecozones.

**Table 5.** Census of the suborder Oribatida, excluding Astigmata (Acari: Acariformes: Sarcoptiformes) in Canada.

Taxon^1^	No. species reported in Lindquist et al. (1979)	No. species currently known from Canada	No. BINs^2^ available for Canadian species	Est. no. undescribed or unrecorded species in Canada	General distribution by ecozone^2A^	Information sources
**Infraorder Palaeosomata**						
**Superfamily Acaronychoidea**						
Acaronychidae	0	1	0	0	Pacific Maritime	
Archeonothridae	0	1	0	0	Pacific Maritime	
**Superfamily Palaeacaroidea**						
Palaeacaridae	1	1	0	1	most ecozones	
**Superfamily Ctenacaroidea**						
Adelphacaridae	0	0	0	1	Pacific Maritime	
Aphelacaridae	1	1	0	0	Pacific Maritime, Montane Cordillera, Prairies	
Ctenacaridae	0	0	0	1	Mixedwood Plains	
**Infraorder Enarthronota**						
**Superfamily Brachychthonioidea**						
Brachychthoniidae	25	38	95	57	all ecozones	Marshall et al. 1987, Walter et al. 2014
**Superfamily Atopochthonioidea**						
Atopochthoniidae	1	1	0	0	Pacific and Atlantic Maritime, Boreal Cordillera, Mixedwood Plains	
Pterochthoniidae	1	1	0	0	Pacific Maritime, Boreal Cordillera, Boreal Plains, Mixedwood Plains	
**Superfamily Hypochthonioidea**						
Eniochthoniidae	2	3	8	5	Boreal ecozones and southward	Norton and BehanPelletier 2007
Hypochthoniidae	2	2	4	3	all ecozones	
Lohmanniidae*	0	0	0	1	Mixedwood Plains	
Mesoplophoridae	2^3^	2	1	1	Boreal Cordillera, Prairies, Mixedwood Plains, Atlantic Maritime	Niedbała 2002
**Superfamily Protoplophoroidea**						
Cosmochthoniidae	2^4^	1	0	1	Montane Cordillera, Mixedwood Plains	
Haplochthoniidae	1	0	0	1	Prairies	
Sphaerochthoniidae	0	1	0	1	Prairies	
**Superfamily Heterochthonioidea**						
Arborichthoniidae	0	1	0	0	Mixedwood Plains	Norton 1982
Trichthoniidae	?^4^	1	0	0	Boreal Shield, Mixedwood Plains, Atlantic Maritime, Newfoundland Boreal	Marshall and Reeves 1971
**Infraorder Parhyposomata**						
**Superfamily Parhypochthonioidea**						
Gehypochthoniidae	1	2	0	1	Pacific Maritime, Taiga Cordillera, Arctic, Boreal Shield, Mixedwood Plains	
Parhypochthoniidae	1	2	0	1	Pacific and Atlantic Maritime, Boreal Plains, Prairies, Mixedwood Plains	
**Infraorder Mixonomata**						
**Superfamily Eulohmannioidea**						
Eulohmanniidae	1	1	0	1	Pacific and Atlantic Maritime, Arctic, Taiga Cordillera, Mixedwood Plains	
**Superfamily Perlohmannioidea**						
Perlohmanniidae	0	1	2	2	Pacific and Atlantic Maritime, Arctic, Taiga Cordillera	
**Superfamily Epilohmannioidea**						
Epilohmanniidae	1	1	2	2	Pacific Maritime, Taiga Cordillera, Prairies, Mixedwood Plains	
**Superfamily Euphthiracaroidea**						Niedbała 2002
Euphthiracaridae	6	19	30	17	all ecozones	
Oribotritiidae	4	13	11	7	Taiga ecozones and southward	
Synichotritiidae	0	2	4	4	Pacific Maritime	
**Superfamily Phthiracaroidea**						
Phthiracaridae	10	28	68	37	all ecozones	Niedbała 2002
**Infraorder Desmonomata**						
**Hyporder Nothrina**						
**Superfamily Crotonioidea**						
Crotoniidae	12^5^	24	70	25	all ecozones	Colloff 1993
Hermanniidae	3	5	7	4	Pacific and Atlantic Maritime, Arctic	
Malaconothridae	3	3	22	13	all ecozones	Marshall et al. 1987
Nanhermanniidae	2	3	4	4	most ecozones	
Nothridae	5	10	41	22	all ecozones	Marshall et al. 1987, Walter et al. 2014
Trhypochthoniidae	4	7	29	10	all ecozones	Norton et al. 1988, 1996, BehanPelletier and Bissett 1994, Walter et al. 2014
**Hyporder Brachypylina**						
**Superfamily Hermannielloidea**						
Hermanniellidae	3	5	25	13	Boreal ecozones and southward	
Plasmobatidae	0	0	0	1	Mixedwood Plains	
**Superfamily Neoliodoidea**						
Neoliodidae ^6^	4	2	9	5	Boreal ecozones and southward	
**Superfamily Plateremaeoidea**						
Gymnodamaeidae	7	11	40	23	all ecozones	Walter 2009a
Licnodamaeidae	1	0	1	1	Pacific and Atlantic Maritime	
Plateremaeidae	0	0	0	1	Mixedwood Plains, Boreal Shield	
**Superfamily Damaeoidea**						
Damaeidae	24^7,8^	23	77	54	all ecozones	BehanPelletier and Norton 1983, 1985
**Superfamily Eutegaeoidea**						
Anderemaeidae	1	0	4	1	Pacific and Atlantic Maritime, Mixedwood Plains	
Compactozetidae ^9^	5	9	23	14	all ecozones	Walter et al. 2014
**Superfamily Polypterozetoidea**						
Nodocepheidae	0	1	0	0	Pacific Maritime	
Podopterotegaeidae	0	1	0	0	Boreal Shield, Atlantic Maritime, Newfoundland Boreal	
Polypterozetidae	0	0	0	1	Boreal Cordillera	
**Superfamily Microzetoidea**						
Microzetidae	0	1	0	1	Pacific Maritime, Mixedwood Plains	
**Superfamily Ameroidea**						
Ameridae	0	1	0	0	Mixedwood Plains	Chen et al. 2004
Caleremaeidae	?^8^	2	3	3	Pacific Maritime, Cordillera ecozones, Arctic, Newfoundland Boreal	
Damaeolidae	1	1	0	1	Pacific Maritime, Boreal Plains, Mixedwood Plains	
Eremobelbidae	1	1	9	9	Mixedwood Plains	
Eremulidae	1	1	3	3	Mixedwood Plains	
Hungarobelbidae	0	0	0	3	Pacific and Atlantic Maritime, Montane Cordillera	
**Superfamily Zetorchestoidea**						
Eremaeidae	5	32	89	30	all ecozones	BehanPelletier 1993
Megeremaeidae	1	5	3	3	Pacific Maritime, Cordillera ecozones, Boreal Shield, Mixedwood Plains	BehanPelletier 1990
Zetorchestidae	0	0	0	1	Mixedwood Plains	
**Superfamily Gustavioidea**						
Astegistidae	3	5	21	12	all ecozones	Marshall et al. 1987
Gustaviidae	1	1	8	5	Pacific Maritime, Boreal Plains, Prairies, Mixedwood Plains	
Kodiakellidae	0	1	0	0	Pacific Maritime	
Liacaridae	7^10^	8	36	25	all ecozones	
Peloppiidae ^11^	8	17	66	35	all ecozones	Lindo et al. 2010, Lindo 2011, 2015, 2018
Tenuialidae	3	2	11	7	Boreal ecozones and southward	
**Superfamily Carabodoidea**						
Carabodidae	4	16	32	12	all ecozones	Reeves 1992, Reeves and BehanPelletier 1998
**Superfamily Oppioidea**						
Autognetidae	3	5	9	7	all ecozones	BehanPelletier 2015
Machuellidae	0	0	0	1	Mixedwood Plains	
Oppiidae	20^12^	16	144	60	all ecozones	Marshall et al. 1987, BehanPelletier 2010, Walter et al. 2014
Quadroppiidae	?^12^	3	9	5	all ecozones	
Thyrisomidae	5	7	6	5	all ecozones	Marshall et al. 1987, BehanPelletier 2010, Walter et al. 2014
**Superfamily Trizetoidea**						
Suctobelbidae	25	13	70	48	all ecozones	Marshall et al. 1987
**Superfamily Tectocepheoidea**						
Tectocepheidae	3	2	131	30	all ecozones	
**Superfamily Limnozetoidea**						
Hydrozetidae	2	4	8	6	most ecozones	
Limnozetidae	1	10	7	7	most ecozones	BehanPelletier 1989b
**Superfamily Ameronothroidea**						
Ameronothridae ^13^	3	4	5	3	Boreal Plains, Arctic, Newfoundland Boreal	Marshall et al. 1987
Selenoribatidae	0	0	0	1	Pacific Maritime	
Tegeocranellidae	0	1	0	1	Mixedwood Plains, Atlantic Maritime	BehanPelletier 1997b
**Superfamily Cymbaeremaeoidea**						
Cymbaeremaeidae	1	8	36	18	all ecozones	BehanPelletier 1987, 1989a, Norton et al. 2010
**Superfamily Licneremaeoidea**						
Dendroeremaeidae	0	1	4	4	Pacific Maritime	BehanPelletier et al. 2005
Eremellidae	0	1	2	1	Mixedwood Plains	
Licneremaeidae	0	0	0	1	Pacific Maritime	
Micreremidae	1	1	3	1	Mixedwood Plains, Atlantic Maritime	
Passalozetidae	1	2	2	2	Boreal and Montane Cordilleras, Boreal Plains, Boreal Shield, Prairies	
Scutoverticidae	1	1	7	7	Montane Cordillera, Prairies	
**Superfamily Phenopelopoidea**						
Phenopelopidae ^14^	3	8	85	47	all ecozones	Norton and BehanPelletier 1986
Unduloribatidae	0	1	0	0	Taiga ecozones and southward	BehanPelletier and Walter 2009
**Superfamily Achipterioidea**						
Achipteriidae	4	12	53	20	all ecozones	Lindo et al. 2008
Tegoribatidae	5	6	40	25	all ecozones	BehanPelletier and Walter 2013, BehanPelletier 2017
**Superfamily Oribatelloidea**						
Oribatellidae	12	22	46	23	all ecozones	BehanPelletier 2011, 2013, BehanPelletier and Walter 2012
**Superfamily Oripodoidea**						
Haplozetidae	5	12	39	35	all ecozones	Walter and Latonas 2013
Mochlozetidae	1	2	8	5	Arctic, Prairies, Boreal Plains, Boreal Shield, Mixedwood Plains	
Oribatulidae	26^15^	11	175	92	all ecozones	Marshall et al. 1987, BehanPelletier et al. 2002
Oripodidae	2^16^	1	16	10	Pacific and Atlantic Maritime, Prairies, Boreal Shield, Mixedwood Plains	
Parakalummidae	4	2	37	20	most ecozones	
Scheloribatidae	?^15,16^	13	184	93	all ecozones	Marshall et al. 1987, BehanPelletier et al. 2002, Knee 2017b
**Superfamily Ceratozetoidea**						
Ceratokalummidae	0	0	7	4	Boreal Cordillera, Mixedwood Plains	
Ceratozetidae	35^17^	53	245	70	all ecozones	BehanPelletier 1984, 1985, 1986, 2000, BehanPelletier and Eamer 2009
Chamobatidae	1	1	40	21	Taiga ecozones and southward	
Euzetidae	0	0	2	2	Boreal Plains, Boreal Shield, Mixedwood Plains, Atlantic Maritime	
Humerobatidae	?^17^	2	2	2	Pacific and Atlantic Maritime, Boreal Cordillera, Mixedwood Plains	Marshall et al. 1987
Punctoribatidae ^18^	10	35	35	30	all ecozones	BehanPelletier 1988, 1994, BehanPelletier et al. 2001, BehanPelletier and Eamer 2005, 2008
Zetomimidae	1	5	21	21	Taiga ecozones and southward	BehanPelletier 1996, BehanPelletier and Eamer 2003
**Superfamily Galumnoidea**						
Galumnidae	8	5	93	52	all ecozones	Marshall et al. 1987
**Total**	**354**	**592**	**2429**	**1267**		

*This family is anticipated for Canada although no specimens have been collected to date; for other families with 0 known species, some undetermined material has been collected in Canada. **^1^**Classification follows Schatz et al. 2011, with the addition of modifications by Walter and Latonas (2009) for Haplozetidae; BehanPelletier (2013) for Oribatellidae; Norton and Ermilov (2014) for Eutegaeoidea; BehanPelletier (2017) for Tegoribatidae. **^2^**Barcode Index Number, as defined in Ratnasingham and Hebert (2013). **^2A^**See figure 1 in Langor (2019) for a map of ecozones. **^3^**Includes former Archoplophoridae. **^4^***Trichthonius* previously included in Cosmochthoniidae, now in Trichthoniidae. **^5^**Includes former Camisiidae. **^6^**As Liodidae in Lindquist et al. (1979). **^7^**Includes former Belbidae and parts of Belbodamaeidae. **^8^**Caleremaeidae includes parts of former Belbodamaeidae (*Veloppia*). **^9^**As Cepheidae in Lindquist et al. (1979). **^10^**Includes former Xenillidae. **^11^**As Metrioppiidae in Lindquist et al. (1979). **^12^**Quadroppiidae was included in Oppiidae. **^13^**Includes former Podacaridae. **^14^**As Pelopidae in Lindquist et al. (1979). **^15^**Scheloribatidae was included in Oribatulidae. **^16^***Parapirnodus* previously included in Oripodidae, now in Scheloribatidae. **^17^***Humerobates* previously included in Ceratozetidae, now in Humerobatidae. **^18^**As Mycobatidae in Lindquist et al. (1979).

### Hyporder Astigmata

The Astigmata originated within the oribatid lineage, Desmonomata, and then radiated morphologically and ecologically, adapting to a remarkable diversity of niches (OConnor 2009). Adult astigmatans are similar in appearance to immature oribatids, and hence this clade is thought to have arisen via neoteny. Their evolutionary success can be attributed in part to the evolution of a non-feeding deutonymphal stage for dispersal and for enduring adverse environmental conditions. These traits facilitated colonization of the patchy, transient habitats in which free-living astigmatans thrive, such as animal nests, decaying wood, carrion, fungal sporocarps, and stored products. Many species disperse on insects and have developed relationships with them beyond simple phoresy. Similarly, several lineages of Astigmata have evolved commensal and parasitic relationships with vertebrates, living on and in the skin, fur and feathers of mammals and birds (Psoroptidia: Sarcoptoidea and feather mites). Most non-parasitic Astigmata are assumed to be fungivorous or saprophagous, though vertebrate commensals may also feed on sebaceous oils (OConnor 2009). In Canada, we now know of 441 species of Astigmata from 43 families, compared to a mere 142 species in 1979 (Table 6). In addition, three other families are recorded in Canada, based on unidentified material. The concepts of several families and their placement in superfamilies have changed substantially since 1979. Canestriniidae (Canestrinioidea) was predicted to occur in Canada by Lindquist et al. (1979); we do not expect it to occur here, with the nearest known locality being in the southeastern USA (B OConnor pers. comm.).

The most notable improvements in taxonomy of Astigmata in Canada are for mites associated with the plumage and skin of birds (Analgoidea, Pterolichoidea) (Table 1). There are significant, mostly unpublished, additions in other families, especially Acaridae and Chirodiscidae. The case of feather mites demonstrates how little is known and published and how much work is left to do for the taxonomy of Astigmata in Canada: Galloway et al. (2014) published 118 new records for Canada and mentioned 38 undescribed species of feather mites from grassland birds in Alberta and Manitoba alone! Molecular work conducted for Astigmata in Canada has been relatively limited, with only 153 BINs generated from 19 families; most of these have not yet been confidently assigned to named species. The Acaridae is well represented, encompassing about one third of BINs for Astigmata. In contrast, many families are poorly represented by BINs, compared to the diversity of known or anticipated species. This may be in part due to the limited sampling of Astigmata, which typically live as symbionts of vertebrates or in cryptic, patchy habitats. We estimate that over 1600 species of Astigmata occur in Canada, most of which (>70%) are yet to be discovered.

### Superfamily Histiostomatoidea

The one family in Canada, Histiostomatidae, consists of filter-feeding microbivores associated with moist decaying substrates and aquatic microhabitats. In Canada, they have been found associated with decaying bulbs, pitcher plants, insect and worm cultures, as well as with insects such as bark beetles, bees, carrion- and dung-breeding flies on which they disperse. New records since 1979 include two new species phoretic on amphipods (Fain and Colloff 1990) and one described from sphaerocerid flies (Samsinak 1989). Only 21 named species are known from Canada, but four times that many are unrecorded or undescribed (Table 6).

### Superfamily Hemisarcoptoidea

Five families of Hemisarcoptoidea have described species recorded in Canada (Table 6). This group includes fungivores in stored products (Carpoglyphidae, Hemisarcoptidae, Winterschmidtiidae), on foliage and in bark crevices (Winterschmidtiidae), algivores in littoral habitats (Hyadesiidae), parasites of coccinellid beetles and scale insects (Hemisarcoptidae), and various associates of subcortical beetles (OConnor 2009). Some winterschmidtiids (subfamily Ensliniellinae) are obligate associates of Hymenoptera, and include a species described from leafcutter bees (Megachilidae) in Nova Scotia which feeds on moulds growing on pieces of leaves in the bee nests (OConnor and Eickwort 1988; Table 6). A recent revision of Chaetodactylidae has three new Canadian records of obligate cleptoparasites or parasitoids of leafcutter bees, including two new species (Klimov and OConnor 2008). In total, 17 hemisarcoptoid species are known from Canada and over 50 more species in these five families are expected (Table 6). A sixth family, Algophagidae, has at least one unidentified species in Canada, and a few representatives in the neighbouring USA from water-filled treeholes or tree sap flux likely occur here.

### Superfamily Glycyphagoidea

Ancestrally associated with vertebrate nests, this superfamily includes numerous nest-dwelling fungivore-detritivores, as well as forms that evolved parasitism in the hair follicles of their mammalian hosts (Chortoglyphidae, Echimyopodidae). In addition to inhabiting nests, they can be found on mammals (more rarely birds) or insects on which they are phoretic, as well as in litter and anthropogenic habitats such as house dust and stored products (a few Glycyphagidae, Aeroglyphidae, Chortoglyphidae) (OConnor 2009).

Three families have a total of 24 recorded species in Canada, including several new records of glycyphagids and chortoglyphids from rodent hosts such as muskrats, beavers and mountain beavers since 1979 (Table 6). Rosensteiniidae, associated with bats and their guano, is newly recorded from southern Canada, but as an undetermined species. Echimyopodidae and Euglycyphagidae are anticipated for Canada. Overall, an additional 32 species of glycyphagoids are expected for Canada (Table 6).

### Superfamily Acaroidea

The family Acaridae is the most diverse family within the Acaroidea at both the global and national levels. It is agriculturally the most important group of astigmatic mites because of their role as pests of bulbs, greenhouse vegetables, and stored products (e.g., grains, cheese, meat), where they can feed directly on the cereal grain itself or on green plant tissues (Sinha 1979, Beaulieu and Knee 2014). Most acarid mites are fungivore-saprophages and breed where fungi and other microbes abound, including soil and patchy habitats such as compost, rotting wood, tree bark, bracket fungi, phytotelmata, beetle galleries, and hymenopteran and avian nests. Some Acaridae and Suidasiidae feed on pollen inside bee nests in Canada. There are only a small number of scattered Canadian records of Acaridae and other Acaroidea published since 1979 (often identified only to genus level). Over 40 additional species are expected in Canada, mostly of Acaridae (Table 6). One family, Gaudiellidae, associated with bumble bees, has been newly recorded since 1979. The superfamily is in dire need of revision.

### Superfamily Hypoderatoidea

The sole family, Hypoderatidae, comprises parasites of birds and desert-dwelling rodents (OConnor 2009). This family was not recorded from Canada in 1979 but since then one species has been recorded from the skin of a kingfisher in Ontario (Pence and Gray 1996). Many of the species so far recorded from adjacent states of the USA are anticipated to occur in Canada on the same bird hosts (Table 6).

### Superfamily Sarcoptoidea

This group includes ‘fur mites’ (Chirodiscidae, Listrophoridae, Atopomelidae) which live in the fur of mammals and feed on sebaceous materials (OConnor 2009, Bochkov 2011). Other sarcoptoids obtain fluids from the skin surface (Myocoptidae, Psoroptidae), live in hair follicles (Rhyncoptidae), or burrow in the skin (Sarcoptidae) of their mammalian hosts. More invasive forms colonize the ears of various mammals (some Psoroptidae), nasal passages and lungs of rodents (Gastronyssidae, Pneumocoptidae), or the eye orbits and stomach of bats (Gastronyssidae). The effects of these mites on their hosts vary from relatively benign, such as the irritation caused by ear mites (Psoroptidae: *Otodectes*) of domestic animals, to highly detrimental, such as sarcoptic mange (caused by *Sarcoptesscabiei* [L.]) which can cause skin hyperkeratosis and hair loss and lead to population declines in domestic and wild mammals (OConnor 2009, Murray and St Clair 2017).

Five of the 12 globally recognized families of Sarcoptoidea are known in Canada and four others are anticipated to occur (Table 6). Currently, 38 species are recorded from Canada, more than double that recorded in 1979. Chirodiscidae experienced the largest increase since 1979, with 10 of 11 species currently recorded being restricted to beavers (Table 6; CNC). Eight more species of *Schizocarpus* may occur on beavers in Canada (Fain and Whitaker 1988, Bochkov 2010). Other new Canadian records since 1979 are from mustelids (Chirodiscidae), small rodents (Myocoptidae, Listrophoridae), rabbits, and bovid and cervid hosts (Psoroptidae). Representatives of four families not yet recorded in Canada are expected to occur here, based on nearby USA records: Atopomelidae, Rhyncoptidae, Pneumocoptidae, and Gastronyssidae. Overall, approximately 50 additional species are expected to be found in Canada (Table 6).

### Superfamilies Pterolichoidea, Analgoidea

With 280 species from 25 families recorded (Table 6), these two superfamilies represent almost two thirds of all known species and more than half of the families of Astigmata in Canada. They dominate the diversity of acarofauna living on birds, with up to 10 species recorded from a given host species in Canada (SV Mironov, HC Proctor, and TD Galloway unpubl. data). Most families consist of the true “feather mites”, which are mostly paraphagous commensals, feeding on uropygial gland secretions, as well as on fungi and other organic material (algae, pollen) found on the feathers (Proctor 2003, OConnor 2009). A few families include true parasites that feed on or in the skin (Dermationidae, Epidermoptidae, Knemidocoptidae, Laminosioptidae), consume pith inside quills (Ascouracaridae, Dermoglyphidae, Syringobiidae), or eat live tissues in respiratory tracts (Cytoditidae, Turbinoptidae). Some of these mites significantly affect their wild and domestic hosts, causing dermatitis, scaly-leg and -face diseases of birds, or possibly weaken feather quills after feeding on the inner pith (Proctor and Owens 2000, Proctor 2003). Pyroglyphid mites are unique among the Analgoidea in that most species live as scavengers in the nests of their hosts (birds and mammals) rather than on their bodies, eating skin flakes, hair, and other organic debris. Some pyroglyphids (*Dermatophagoides* spp. and other dust mites) have adapted to living in houses where they feed on shed human skin that has been colonized by fungi, and are the culprits behind many respiratory allergies (Colloff 2009). Taxonomic knowledge of the two superfamilies in Canada has grown an order of magnitude since 1979, thanks to the work of Mironov, Proctor, and Galloway. The known fauna increased from a mere two to 65 species of Pterolichoidea and from 19 to 215 species of Analgoidea, with 12 of the 25 families in Canada representing new records for the country (Table 6).

Astigmatan feather mites, nasal mites, skin and quill mites are now recorded from at least 252 of the 690 bird species occurring in Canada (Lepage 2018), with a sampling bias for birds of the Prairie Provinces where most of the relevant research has been conducted (Galloway et al. 2014; Mironov, Proctor and Galloway unpubl. data). Most bird families that occur in Canada have been sampled for mites. Mironov, Proctor and Galloway have aimed for taxonomic breadth of hosts, which should parallel taxonomic breadth of mites. Most members of Analgoidea and Pterolichoidea known from Canada have been collected by washing the bodies of birds that were either found dead or that died in wildlife rehabilitation centres rather than by targeted collecting from particular species of birds. The most notable increase among families is for the world’s largest family of feather mites, Proctophyllodidae, the species of which inhabit wing feathers of passerines and hummingbirds (Galloway et al. 2014; Table 6). This is followed by the Analgidae, which inhabit downy feathers (Mironov and Galloway 2002). Next are the Alloptidae and Avenzoariidae, mostly associated with various aquatic birds (OConnor 2009; Table 6). The only Ascouracaridae recorded is a potentially host-specific quill mite collected from a threatened bird in Canada, the whip-poor-will (*Caprimulgusvociferus* Wilson). Families of mites that live on or in skin or in quills may be underrepresented due to sampling bias. Adult females of some species of Epidermoptidae are hyperparasitic on hippoboscid flies or lice and can be collected from these hosts, including two species recently reported from Canada (Knee and Galloway 2017a, Goater et al. 2018; Table 6). The family Pyroglyphidae may also be underrepresented since nests are less often targeted than birds.

Five additional families are anticipated in Canada (Table 6): Gaudoglyphidae, represented by one species living in the quills of domestic chickens (Bruce and Johnston 1976); Laminosioptidae, subcutaneous and follicular parasites (Skoracki et al. 2014); Apionacaridae, quill mites (Mironov 2001); Ptyssalgidae, hummingbird-specific quill mites (Atyeo and Gaud 1979); and Cytoditidae, inhabiting the nasal cavity, lungs or air sacs of birds. Based on a number of undescribed species at hand as well as their prevalence, level of host specificity observed so far, and the potential number of host species, we anticipate ~900 additional species of analgoids and pterolichoids on birds in Canada (Table 6).

**Table 6.** Census of the hyporder Astigmata (Acari: Acariformes: Sarcoptiformes: Oribatida) in Canada.

Taxon^1^	No. species reported in Lindquist et al. (1979)	No. species currently known from Canada	No. BINs^2^ available for Canadian species	Est. no. undescribed or unrecorded species in Canada	General distribution by ecozone^2A^ and vertebrate host range	Information sources
**Superfamily Histiostomatoidea**						
Histiostomatidae ^3^	20	21	17	80	all ecozones	Fain and Colloff 1990, Walter and Latonas 2012
**Superfamily Hemisarcoptoidea**						
Algophagidae	0	0	0	3	Boreal ecozones and southward	Fashing and Wiseman 1980
Carpoglyphidae	1	1	0	1	Boreal ecozones and southward	Baker and Delfinado 1978, Beaulieu and Knee 2014
Chaetodactylidae	1	3	1	3	Taiga Plains, Mixedwood Plains	Klimov and OConnor 2008
Hemisarcoptidae	1	3	19	25	Boreal ecozones and southward	Parent 1979, OConnor and Houck 1989
Hyadesiidae	1	1	0	5	Pacific and Atlantic Maritime, Newfoundland Boreal	Fain and Ganning 1989
Winterschmidtiidae	15^4^	9	2	20	all ecozones	OConnor and Eickwort 1988, Walter and Latonas 2012, Beaulieu and Knee 2014
**Superfamily Glycyphagoidea**						
Aeroglyphidae	?^5^	1	0	0	Boreal ecozones and southward	Beaulieu and Knee 2014
Chortoglyphidae	1	3	0	4	Taiga ecozones and southward; rodents	Fain and Spicka 1977, Krantz et al. 2003, Whitaker et al. 2007
Echimyopodidae*	1	0	0	3	Taiga ecozones and southward; squirrels	Fain and Philips 1977
Euglycyphagidae*	0	0	0	1	Mixedwood Plains; bird nests	Fain and Philips 1977
Glycyphagidae	17^5,6^	20	0	20	all ecozones; small mammals	Whitaker et al. 2007, Walter and Latonas 2012, Beaulieu and Knee 2014
Rosensteiniidae	0	0	0	4	Taiga ecozones and southward; bats	Dood and Rockett 1985
**Superfamily Acaroidea**						Walter and Latonas 2012
Acaridae	45^7^	56	58	40	all ecozones	Beaulieu and Knee 2014
Gaudiellidae	0	1	0	0	Mixedwood Plains	Haas et al. in press
Lardoglyphidae	?^7^	1	0	1	Prairies, Mixedwood Plains	Iverson et al. 1996
Suidasiidae	?^7^	2	0	3	Boreal ecozones and southward	Beaulieu and Knee 2014
**Superfamily Hypoderatoidea**						
Hypoderatidae	0	1	0	15	Taiga ecozones and southward; a few bird orders	Pence and Gray 1996, Pence et al. 1997
**Superfamily Sarcoptoidea**						Whitaker et al. 2007, Bochkov 2010
Atopomelidae*	0	0	0	1	Mixedwood Plains; opossum, nutria	
Chirodiscidae	1	11	0	12	Taiga ecozones and southward; bats, beaver, mustelids	Fain and Whitaker 1988
Gastronyssidae*^8^	0	0	0	4	most ecozones; rodents, bats	Bochkov et al. 2008
Listrophoridae	7	11	0	15	Taiga ecozones and southward; small mammals	Fain and Yunker 1980, Whitaker and French 1982
Myocoptidae	5	8	0	4	all ecozones; small mammals	Whitaker and French 1982
Pneumocoptidae*	0	0	0	3	Taiga ecozones and southward; rodents	Baker 1951, Bochkov et al. 2008
Psoroptidae	3	4	1	1	Taiga ecozones and southward; mammals	
Rhyncoptidae*^9^	0	0	0	2	Taiga ecozones and southward; bears, raccoons	Fain and Wilson 1979, Yunker et al. 1980
Sarcoptidae	2	4	1	6	all ecozones; bats, other mammals	
**Superfamily Pterolichoidea**						Galloway et al. 2014
Ascouracaridae	0	1	0	10	Boreal ecozones and southward; caprimulgiform birds	Gaud and Atyeo 1996
Cheylabididae	0	1	0	1	all ecozones; accipitriform birds	Peterson et al. 1980
Eustathiidae	0	2	0	10	Boreal ecozones and southward; caprimulgiform birds	Peterson et al. 1980
Falculiferidae	0	3	0	15	Boreal ecozones and southward; columbiform birds	
Freyanidae	0	8	0	40	all ecozones; anseriform and pelecaniform birds	Buscher 1965, Bourgeois and Threlfall 1981
Gabuciniidae	0	7	0	25	all ecozones; accipitriform, caprimulgiform, falconiform and passerine birds	Mironov et al. 2007
Kramerellidae	0	10	2	15	all ecozones; several bird orders	Atyeo and Philips 1984
Pterolichidae	2	19	0	70	all ecozones; several bird orders	Dabert and Ehrnsberger 1999
Ptiloxenidae	0	5	0	5	all ecozones; charadriiform birds	
Rectijanuidae	0	0	0	15	Boreal ecozones and southward; anseriform birds	
Syringobiidae	0	9	0	30	all ecozones; charadriiform birds	Dabert and Ehrnsberger 1995
**Superfamily Analgoidea**						Galloway et al. 2014
Alloptidae	3	22	1	60	all ecozones; several aquatic bird orders	Peterson 1971
Analgidae	1	40	13	90	all ecozones; several bird orders	Wheeler and Threlfall 1986
Apionacaridae*	0	0	0	5	all ecozones; quail (Odontophoridae) and charadriiform birds	
Avenzoariidae	3^10^	27	1	80	all ecozones; several aquatic bird orders and osprey	Ballard and Ring 1979
Cytoditidae*	0	0	0	5	Taiga ecozones and southward; several bird orders	Pence 1975
Dermationidae	?^11^	2	1	20	all ecozones; apodiform, charadriiform, passerine and piciform birds	Mironov et al. 2005
Dermoglyphidae	0	1	0	20	all ecozones; several bird orders	
Epidermoptidae	2^11^	7	1	10	all ecozones; several bird orders	Mironov et al. 2005, Knee and Galloway 2017a, Goater et al. 2018
Gaudoglyphidae*	0	0	0	1	all ecozones; domestic chicken	Bruce and Johnston 1976
Knemidokoptidae ^12^	0	2	0	10	all ecozones; passerine birds	Mironov et al. 2005, Knee and Proctor 2006
Laminosioptidae*	0	0	0	20	all ecozones; several bird orders	Lukoschus and Lombert 1980, Skoracki et al. 2014
Proctophyllodidae	3	61	22	150	all ecozones; mostly passerines with a few exceptions	Wheeler and Threlfall 1986, Valim and Hernandes 2010
Psoroptoididae	0	4	1	40	all ecozones; owls, and gruiform and passerine birds	Wheeler and Threlfall 1986, Mironov 2011
Pteronyssidae	?^10^	20	3	30	all ecozones; apodiform, charadriiform, passerine and piciform birds	Mironov and Galloway 2006
Ptyssalgidae*	0	0	0	5	Boreal ecozones and southward; hummingbirds	
Pyroglyphidae	5	4	1	5	all ecozones; bird and mammal nests	Gaud and Atyeo 1996
Trouessartiidae	0	8	7	50	all ecozones; passerine birds	Wheeler and Threlfall 1986
Turbinoptidae	1	2	0	10	Taiga ecozones and southward; accipitriform and charadriiform birds	Pence 1975, Knee and Galloway 2017b
Xolalgidae	1	15	1	50	all ecozones; several bird orders	
**Unplaced family**						
Heterocoptidae*	0	0	0	1	Mixedwood Plains	
**Total**	**142**	**441**	**153**	**1174**		

*These families are anticipated for Canada although no specimens have been collected to date; for other families with 0 known species, some undetermined material has been collected in Canada. **^1^**Classification mostly follows OConnor (2009), as slightly modified by Schatz et al. (2011), with exception mentioned in footnote 12. **^2^**Barcode Index Number, as defined in Ratnasingham and Hebert (2013). **^2A^**See figure 1 in Langor (2019) for a map of ecozones. **^3^**As Anoetidae in Lindquist et al. (1979). **^4^**Includes former Saproglyphidae. **^5^**Aeroglyphidae was included in Glycyphagidae. **^6^**Includes former Labidophoridae, Fusacaridae and Ctenoglyphidae. **^7^**Lardoglyphidae and Suidasiidae were included in Acaridae. **^8^**As Yunkeracaridae in Lindquist et al. (1979). **^9^**As Audycoptidae in Lindquist et al. (1979). **^10^**Pteronyssidae was previously included in Avenzoariidae. **^11^**Dermationidae was previously included in Epidermoptidae. **^12^**Included in Epidermoptidae in OConnor (2009) and Schatz et al. (2011).

### Heterocoptidae

At least one representative of the unplaced family Heterocoptidae probably occurs in southern Canada, since an undescribed species of the family has been found on a beetle in nearby Michigan (B OConnor pers. comm.). Heterocoptids are presumably paraphagic on their beetle hosts (OConnor 2009).

## Knowledge gaps and opportunities

Although the increase in known species of Acari in Canada is substantial, there are still major taxonomic gaps for all higher mite taxa, except ticks, with the majority of families requiring review or revision in Canada. Even for some of the better known acarine families in North America (e.g., Mesostigmata: Phytoseiidae; Prostigmata: Bdellidae, Tetranychidae, Trombiculidae), there are no species checklists available for Canada. There are large numbers of unpublished records for species present in Canada, but not reported in the world or North American catalogues (Denmark and Evans 2011, Walters et al. 2011, Hernandes et al. 2016, Demite et al. 2018, Migeon and Dorkeld 2018).

Based on the estimated diversity of unrecorded species (Tables 2–6), as well as the need for clarifying species concepts and improving classification, the following taxa are the most notable in requiring revision in Canada. Within the Mesostigmata, it is the Parasitidae, Laelapidae, and families of Uropodoidea, Rhodacaroidea and Ascoidea. Many groups of Prostigmata need particular attention: Cunaxidae, Eupodidae, Tydeoidea, Halacaridae, Erythraeidae, Trombiculidae, Cheyletoidea, Tenuipalpidae, Pygmephoroidea, and Tarsonemidae. Future research priorities in water mites include Hygrobatidae, Lebertiidae and Sperchontidae. The Eriophyoidea, now tentatively in the Endeostigmata, urgently needs revision. The greatest uncertainty in species numbers for Oribatida lies in smaller-bodied and/or highly diverse families (e.g., Brachychthoniidae, Oppiidae, Suctobelbidae, Oribatulidae, Scheloribatidae), but also significant efforts for revisions are required within the Damaeidae, Liacaridae, and Galumnidae. Furthermore, despite considerable progress in several families (e.g., Ceratozetidae, Peloppiidae, Eremaeidae), we suspect that large numbers of species remain unrecorded for these families. While feather mites (Analgoidea, Pterolichoidea) are undergoing major, needed revisions, other Astigmata that require particular focus are Acaridae, Histiostomatidae and Glycyphagidae.

We anticipate an additional 29 families of mites in Canada (indicated by * in Tables 2–6): Mesostigmata (4 families), Trombidiformes (10), Oribatida (1), Astigmata (12) and Endeostigmata (2). Based on our estimates (Table 1), the majority of all species of Mesostigmata (60%), Trombidiformes (66%), Endeostigmata (86%; due mostly to Eriophyoidea), Oribatida (68%), and Astigmata (73%) are yet to be discovered, identified or described in Canada. This represents at least six times the number of species newly recorded in Canada in the ~40 years since Lindquist et al. (1979)!

Many habitats are vastly underexplored for mites, often because the habitat is cryptic, or hard to reach physically. In particular, we anticipate new species discoveries in the following habitats: deep soils; arboreal, littoral and alpine habitats; groundwater and hyporheic zones of fresh water; all marine habitats; cave system substrates; angiosperm hosts, including their flowers; patchy habitats such as dung, dead wood, fungal sporocarps, plant galls, tree sap flows; and vertebrate and invertebrate nests/burrows and their insect residents. Many of the thousands of aquatic and terrestrial invertebrate species in Canada, particularly moths, dipterans, coleopterans, hymenopterans, but also millipedes, spiders, amphipods and gastropods are hosts to commensal and parasitic mites that are expected to occur in Canada but have not yet been recorded. Among vertebrate hosts, birds are now better targeted (Galloway et al. 2014), but surveys for mites on reptiles, amphibians and especially mammals, including bats and aquatic species (e.g., pinnipeds), are relatively rare in Canada. Overall, there is a dearth of information on the distribution, habitat, host associations, feeding biology, life history and immature stages of most species of Acari in Canada.

Addressing the deficiency in taxonomic and faunistic knowledge of Acari in Canada will require major efforts towards the following: (1) targeted exploration of many more habitats and hosts across all ecozones; (2) integrated approaches combining morphological, molecular and ecological data to elucidate species boundaries, including for cryptic species; and (3) species-level assessments and confirmation for taxa collected during biological surveys and ecological studies (Gotelli 2004). Beyond such taxonomic endeavours, studies targeting the feeding habits as well as host relationships of selected species or assemblages are necessary to refine our understanding of the diverse roles of mites in Canadian ecosystems. Finally, “to fulfill our national and worldwide duties of classifying and describing…species, particularly to address agricultural (pests, invasive species) problems before they get out of hand (or to be ready when they arise), and to address conservation and biodiversity questions before the biodiversity disappears” (Lumley et al. 2013), we will need to enhance and accelerate efforts in acarology. The foremost approach for this will not be to simply maintain, but to increase the level of acarological expertise in the country.

## Contributions of authors

The tables and text for each section were mostly prepared by the following authors: Parasitiformes by F Beaulieu, W Knee, and E Lindquist; Trombidiformes by F Beaulieu, V Nowell, M Schwarzfeld, W Knee, E Lindquist and I Smith; Endeostigmata by F Beaulieu, W Knee and D Walter; Oribatida by Z Lindo, V BehanPelletier and L Lumley; Astigmata by F Beaulieu, W Knee, V Nowell, H Proctor, S Mironov, and T Galloway. Data compilation from the literature and unpublished specimen data was led by the authors mentioned above, with major contributions by W Knee and V Nowell for non-oribatids; feather mite data (Astigmata) were provided by H Proctor, S Mironov, and T Galloway, and water mite data (Trombidiformes: Hydrachnidia) by I Smith. Summer students significantly contributed to specimen databasing (see Acknowledgements). The BIN data have been provided by M Young, and she contributed significantly to text remarks on molecular aspects. E Lindquist, H Proctor, D Walter, and T Galloway contributed significantly to the overall editing of the text. E Lindquist acted as a key mentor to the first three authors during all the main steps of this project.
